# Genetic structure in four West African population groups

**DOI:** 10.1186/1471-2156-6-38

**Published:** 2005-06-24

**Authors:** Adebowale A Adeyemo, Guanjie Chen, Yuanxiu Chen, Charles Rotimi

**Affiliations:** 1 College of Medicine, University of Ibadan, Ibadan. Nigeria; 2 National Human Genome Center, Howard University, Washington DC, USA

## Abstract

**Background:**

Africa contains the most genetically divergent group of continental populations and several studies have reported that African populations show a high degree of population stratification. In this regard, it is important to investigate the potential for population genetic structure or stratification in genetic epidemiology studies involving multiple African populations. The presences of genetic sub-structure, if not properly accounted for, have been reported to lead to spurious association between a putative risk allele and a disease. Within the context of the Africa America Diabetes Mellitus (AADM) Study (a genetic epidemiologic study of type 2 diabetes mellitus in West Africa), we have investigated population structure or stratification in four ethnic groups in two countries (*Akan *and *Gaa-Adangbe *from Ghana, *Yoruba *and *Igbo *from Nigeria) using data from 372 autosomal microsatellite loci typed in 493 unrelated persons (986 chromosomes).

**Results:**

There was no significant population genetic structure in the overall sample. The smallest probability is associated with an inferred cluster of 1 and little of the posterior probability is associated with a higher number of inferred clusters. The distribution of members of the sample to inferred clusters is consistent with this finding; roughly the same proportion of individuals from each group is assigned to each cluster with little variation between the ethnic groups. Analysis of molecular variance (AMOVA) showed that the between-population component of genetic variance is less than 0.1% in contrast to 99.91% for the within population component. Pair-wise genetic distances between the four ethnic groups were also very similar. Nonetheless, the small between-population genetic variance was sufficient to distinguish the two Ghanaian groups from the two Nigerian groups.

**Conclusion:**

There was little evidence for significant population substructure in the four major West African ethnic groups represented in the AADM study sample. Ethnicity apparently did not introduce differential allele frequencies that may affect analysis and interpretation of linkage and association studies. These findings, although not entirely surprising given the geographical proximity of these groups, provide important insights into the genetic relationships between the ethnic groups studied and confirm previous results that showed close genetic relationship between most studied West African groups.

## Background

Africa is inhabited by populations that show high levels of genetic diversity compared to most other continental populations today and it is thought to be the ancestral home of modern humans. African populations have the largest number of population specific autosomal, X-chromosomal and mitochondrial DNA haplotypes with non-African populations having only a subset of the genetic diversity present in Africa [1]. Estimates of F_ST _(the classic measure of population subdivision) from mitochondrial DNA are much higher in Africa than other populations, as summarized by Tishkoff et al [1]. In addition, analyses from studies based on autosomal SNPs, STRPs or Alu elements show higher F_ST _values for African populations [2-4]. Recent studies of world populations based on large genomic data also reported significant population structure among the African groups [5,6]. However, given the cultural and linguistic diversity of African populations (with over 2000 distinct ethnic groups and languages), these studies have typically included only a handful of African populations indicating that most African populations have not been studied. As previously noted, most existing genetic data on African populations have come from a few countries that are relatively economically developed and/or with key research or medical centers [1]. Availability of more genetic data from sub Saharan Africa will clearly be useful in our understanding of population structure, demographic history and the efforts to map disease-causing genes.

Several genetic epidemiologic studies mapping complex disease-causing genes have been designed to take advantage of the population genetic characteristics of contemporary African populations for fine mapping of informative genomic regions. These characteristics include lower linkage disequilibrium values [5-9] and smaller haplotype block sizes [10,11]. On the other hand, African populations have more divergent patterns of LD and more complex pattern of population substructure or stratification [12-17]. Population stratification refers to differences in allele frequencies between cases and controls due to systematic differences in ancestry rather than association of genes with disease and it can have a major impact on the ability of genetic epidemiologic studies to detect valid associations between a putative risk allele and a disease or trait.

We investigated population structure or stratification in four ethnic groups in two countries in West Africa (*Akan *and *Gaa-Adangbe *from Ghana, *Yoruba *and *Igbo *from Nigeria) using data from 372 autosomal microsatellite loci [see Additional file 1] typed in 493 unrelated persons (986 chromosomes). Firstly, we used a clustering algorithm to infer population structure in the whole sample while ignoring ethnic group information and compare our findings to reported ethnic grouping. Next, we used analysis of molecular variance (AMOVA) models on the same data. Finally, we estimate FST and allele sharing distances between all population pairs.

## Results

**Figure 2:**
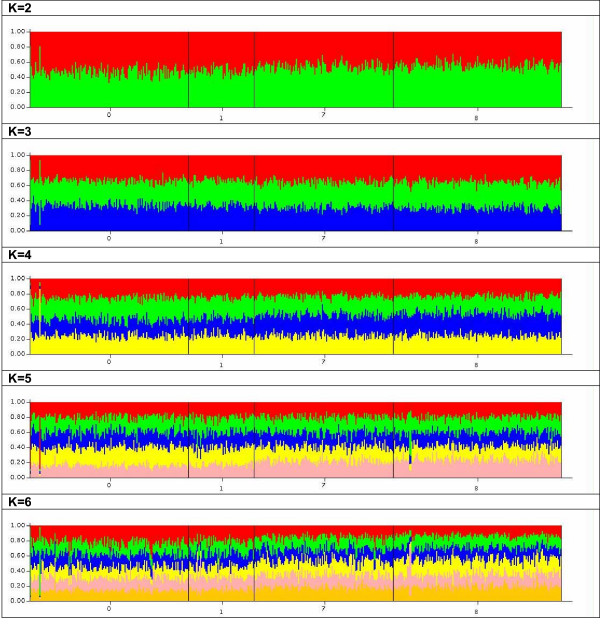
Bar plots of estimates of membership coefficient (Q) for each individual by ethnic group. Legend for population groups: 0 = Akan, 1 = Gaa-Adangbe, 7 = Yoruba, 8 = Igbo. Analyzed under admixture model, assuming correlated allele frequencies.

The estimates of the logarithms of the probability of the data under the models and assumptions regarding independence of allele frequencies are shown in Table 1. Under the admixture model, the smallest probability is associated with a prior K of 1 and little of the posterior probability is associated with higher K values. The distribution of members of the sample to inferred clusters is consistent with this observation. The proportion of individuals assigned to each cluster is approximately the same with little variation between ethnic groups (Table 2). This symmetry is strongly suggestive of the absence of population structure in the AADM study sample. This is so because real population structure is associated with individuals being strongly assigned to one inferred cluster or another with the proportions assigned to each ethnic group showing asymmetry. The posterior probability under the no-admixture model also favours a K of 1. Examination of the distribution of individuals sampled to inferred clusters also shows the same strong symmetry. These consistent displays of symmetry suggest that a K of 1 is the most parsimonious model. The same conclusion was reached by examining the membership coefficients (Q). Irrespective of the value of K between the range of 2 and 6, Q is similar across the whole sample as illustrated by the bar plots in Figure 2.

**Table 1:** Estimates of log probability of data under various assumptions for K = 1–6

K	**No-admixture model**		**Admixture model**	
	
	Log P (X|K)	Posterior probability	Log P (X|K)	Posterior probability
1	-642431	~1.0	-642486	~0.99
2	-646015	0	-642606	5.6 × 10^-57^
3	-649140	0	-642800	2.9 × 10^-137^
4	-649168	0	-644022	0
5	-647275	0	-645623	0
6	-652040	0	-647265	0

**Table 2:** Proportion of membership of each ethnic group in inferred clusters for *K *= 2 to 6 under admixture model with correlated allele frequencies

		**Inferred cluster**					
**Ethnic group**		**1**	**2**	**3**	**4**	**5**	**6**
**K = 2**							
	Akan	0.52	0.48				
	Gaa-Adangbe	0.51	0.49				
	Yoruba	0.46	0.54				
	Igbo	0.44	0.56				

**K = 3**							
	Akan	0.33	0.33	0.34			
	Gaa-Adangbe	0.33	0.33	0.34			
	Yoruba	0.33	0.34	0.31			
	Igbo	0.35	0.35	0.30			

**K = 4**							
	Akan	0.25	0.29	0.21	0.25		
	Gaa-Adangbe	0.25	0.28	0.22	0.25		
	Yoruba	0.23	0.24	0.28	0.25		
	Igbo	0.23	0.23	0.30	0.24		

**K = 5**							
	Akan	0.20	0.20	0.20	0.24	0.16	
	Gaa-Adangbe	0.20	0.21	0.20	0.22	0.17	
	Yoruba	0.20	0.21	0.18	0.19	0.22	
	Igbo	0.19	0.21	0.18	0.18	0.24	

**K = 6**							
	Akan	0.22	0.17	0.17	0.14	0.16	0.14
	Gaa-Adangbe	0.20	0.17	0.16	0.14	0.18	0.15
	Yoruba	0.15	0.16	0.16	0.18	0.17	0.18
	Igbo	0.13	0.16	0.15	0.20	0.17	0.19

Analysis of molecular variance (AMOVA) shows that most of the variance in the sample is attributable to *within-ethnic group *variation (99.91% of the variance) and *between-ethnic group *variation is only 0.09% (Table 3). Locus-by-locus AMOVA shows that this pattern of partitioning of the variance between within-population and between-population variation is consistent across all loci and can be observed on single locus analysis [see Additional file 2]. An AMOVA model that includes "country" as well as "ethnic group" in the model shows that the variance attributable to *between-country *variation was 0.13%, that due to *between-ethnic group *variation was 0.01% and that due to *within-ethnic group *variation was 99.86% (Table 3). The *between-country *genetic variance in this model was significant, suggesting that the two groups from one country can be distinguished from the groups from the other country.

**Table 3:** Analysis of Molecular Variance (AMOVA) results: AADM Study

**Source of variation**	**d.f.**	**Sum of squares**	**Variance components**	**% variation**
Model A:				
Among ethnic groups	3	494.426	0.126 (V_a_)	0.09
Within ethnic group	982	132287.848	134.713 (V_b_)	99.91
Total	985	132782.274	134.839	
Model B:				
Among countries	1	220.117	0.172 (V_a_)	0.13
Among ethnic groups within countries	2	274.309	0.012 (V_b_)	0.01
Within ethnic group	982	132287.848	134.713(V_c_)	99.86
Total	985	132782.274	134.895	

**Figure 3:**
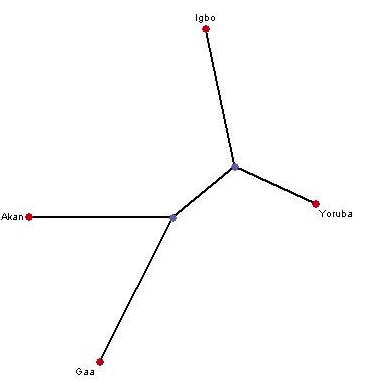
Unrooted radial neighbour-joining tree showing the genetic relationships of the four populations groups studied.

Pair-wise genetic distance measures show that there is little difference between the four ethnic groups (Table 4). The fact that all calculated pair-wise F_ST _values were low suggests little evidence for genetic differentiation between the ethnic groups. The fixation index for the entire sample as estimated by F_ST _is 0.00093. Allele-sharing distances are also similar between the groups (Table 3). Plotting these distances on an unrooted radial tree using a neighbour-joining algorithm (Figure 3) suggests that the two Ghanaian groups can be distinguished from the two Nigerian groups. This observation is consistent with the findings of the hierarchical AMOVA model in Table 3.

**Table 4:** Pairwise genetic distances between the ethnic groups studied

Group	*Akan*	*Gaa-Adangbe*	*Yoruba*	*Igbo*
*Akan*	*	**0.11833**	**0.10410**	**0.10798**
*Gaa-Adangbe*	0.00013	*	**0.12470**	**0.12793**
*Yoruba*	0.00099	0.00072	*	**0.09508**
*Igbo*	0.00177	0.00162	0.00005	*

Notes:

Above diagonal: Allele-sharing distance (Bowcock et al 1994) Below diagonal:

F_ST _(Weir and Cockerham, 1984)

## Discussion

Using data from 372 microsatellite loci typed in 493 unrelated persons from four major ethnic groups in Nigeria and Ghana, we sought for evidence of population structure using several methods. Our results did not show any significant population substructure and no ethnic group corresponded to inferred clusters. This finding has been reported by others [5]. Although Rosenberg et al observed significant population structure among six African groups (*Bantu-Kenya*, *Mandenka*, *Yoruba*, *San*, *Mbuti *Pygmy and *Blaka *Pygmy), they reported that inferred clusters for some of the African populations did not correspond to predefined groups, unlike groups from America, Oceania and Eurasia [5].

The within-population component of genetic variation accounts for most of the diversity in the sample. This is consistent with previous findings [5] showing that the within-population component of genetic variance among six African populations studied was 96.9%; we estimated an even higher value of 99.9% in this study. The higher value of the within-population variance in this study is likely due to the smaller geographic area from which the samples were derived. The maximum distance between any two sites in this study is less than 700 miles and there are no major natural barriers e.g., mountains, between the regions inhabited by the groups. In addition, these four ethnic groups have a long history of trade and other interactions and they all speak languages belonging to the Niger-Kordofanian group. As noted by Cavalli-Sforza et al [18] the genetic relationships observed in West Africa indicate that major migrations and admixtures occurred within the region in earlier times

It is important to point out that despite the small amount of genetic differentiation in the sample as a whole, it was possible to distinguish between the groups from each country using a hierarchical AMOVA model and a dendrogram algorithm. Thus, the absence of significant population structure between the four groups did not mean that the groups could not be distinguished from each other. Rather, the data in Table 4 show that enough differences exist to separate the two populations from Nigeria from those from Ghana.

From the disease-mapping point of view, population stratification is important in the analysis of association genetic data, especially when that data is being used to infer the contribution of genetics to a disease. The presence of undetected population structure can mimic association (leading to more false positives) or mimic lack of association (leading to false negatives) [19]. While there has been much debate about the impact of population stratification on association studies, there are limited data that quantify the magnitude of this effect. The largest study to quantify this effect analyzed data from 11 case-control and case-cohort association studies [20] and showed that there was no statistically significant evidence for stratification. However, most of the studies evaluated above used limited number of markers making it difficult to completely rule out moderate levels of stratification that could lead to the finding of false positive associations.

Typically, efforts are made to minimize the effect of stratification during study design and data analysis, including a careful selection of cases and controls (e.g., matching) and by conducting family-based association tests. However, for the size of study needed to detect typical genetic effects in common diseases, even modest levels of population structure within population groups cannot be safely ignored [19]. Given this, we have searched for evidence of population stratification in this genetic epidemiologic study, the first of its kind for T2DM in West Africa. Noting that the number of markers needed to assess stratification depends on the magnitude of genetic effects under study [19], we have used a large number of markers, rather than just a few dozen as in many studies. The number of markers we have used (372) can bring the conservative 95^th ^percentile upper bound on the level of stratification to within 10% of the true value [20].

## Conclusion

In summary, there was little evidence for significant population substructure in the four major West African ethnic groups represented in the AADM study sample. Classification of individuals into clusters showed symmetry, with roughly the same proportion of each ethnic group assigned to each cluster(s). Ethnicity apparently did not introduce differential allele frequencies that may affect analysis and interpretation of linkage and association studies. These findings, although not entirely surprising given the geographical proximity of these groups, provide important insights into the genetic relationships between the ethnic groups studied and confirm previous results that showed close genetic relationship between most studied West African groups.

## Methods

**Figure 1:**
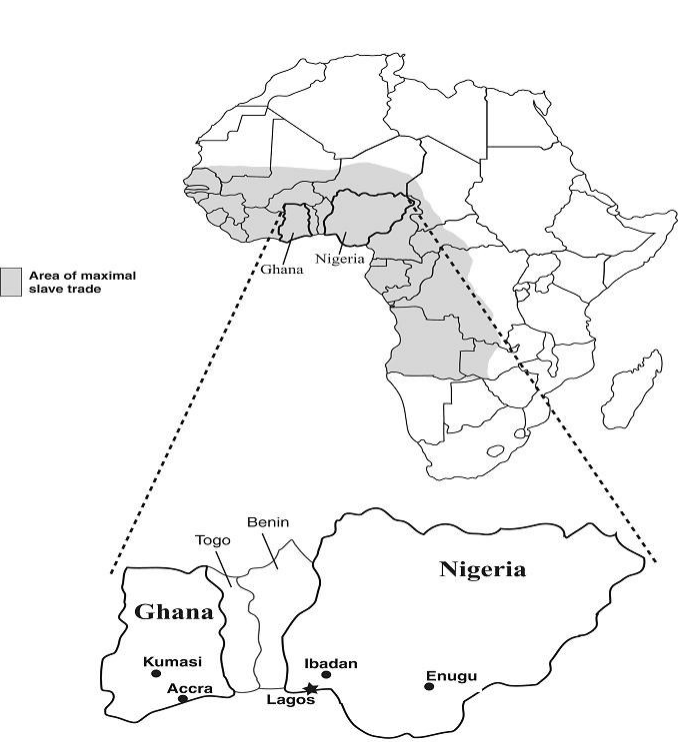
Map of Africa showing the AADM field sites in the two countries.

The AADM study is an affected sibling pair (ASP) design with enrolment of available spouses as controls. Recruitment strategies and eligibility criteria for the families enrolled in this report have been described in a previous publication [21]. The three centers in Nigeria (Enugu, Ibadan and Lagos) enrolled 2 major ethnic groups – *Igbos *(28%) and *Yorubas *(28%); the two centers in Ghana (Accra and Kumasi – see figure 1) enrolled two major ethnic groups – *Akan *(25%) and *Gaa-Adangbe *(11%). For this analysis, 493 unrelated persons were studied, comprising 147 *Akan*, 61 *Gaa-Adangbe*, 129 *Yoruba *and 156 *Igbo *participants.

### Marker set

Genotyping was done at the Center for Inherited Disease Research (CIDR). The CIDR marker set is composed primarily of trinucleotide and tetranucleotide repeats and consists of 392 primer pairs with average spacing of 8.9 cM throughout the genome. There are no gaps in the map larger than 18 cM. The average marker heterozygosity is 0.76. Approximately 10% of the marker loci are different between the current CIDR marker set and the Marshfield Genetics screening set version 8. Almost all reverse primer sequences have been modified from the version 8 sequences in order to reduce '+A' artifacts. The resulting PCR products are sized using a capillary sequencing platform. Data for the markers are generated with 218 PCR reactions (41 triplex reactions, 92 duplex reactions and 85 single reactions). Each primer pair has undergone extensive optimization to improve performance and reliability. Error rate was 0.1% per genotype. Inconsistency rate was 0.11%. Extensive quality checks were carried out to verify consistency of marker genotyping as previously described [22].

For this analysis, all 372 typed autosomal microsatellite markers were included. The markers comprised 272 (73%) tetranucleotide, 46 (12%) trinucleotide and 54 (15%) dinucleotide microsatellites. The markers and their characteristics are provided [see Additional file 1]. The raw genotype data can be obtained by contacting the authors (aadeyemo@howard.edu or crotimi@howard.edu.)

### Analysis

We used a model-based clustering method for inferring population using genotype data consisting of unlinked markers as implemented in the *structure *program version 2.1 [23]. The model assumes there are *K *populations (where *K *may be unknown), each of which is characterized by a set of allele frequencies at each locus. Individuals in the sample are assigned probabilistically to populations, or jointly to two or more populations if their genotypes indicate they are admixed. It is assumed that within populations, the loci are at HWE and linkage equilibrium. This method has the advantage that it does not assume any particular mutation model and it can be applied to microsatellite, SNP and RFLP data. The data was analyzed under an admixture model, assuming correlated allele frequencies between populations as previously described [24]; these assumptions have the advantage of being able to detect recent population divergence and recent admixture, thus giving better performance on difficult problems, although at the potential cost of overestimating K [23]. The analysis was then repeated under a no-admixture model, assuming independence of allele frequencies. Each run was done for K = 1 to 6 after 100,000 burn-in iterations and 10^6 ^estimation iterations (admixture model) or 2 × 10^6 ^estimation iterations (non-admixture model). Each run was carried out several times to ensure consistency of the results. Posterior probabilities for each K were computed for each set of runs.

Analysis of molecular variance (AMOVA) was done using data from all 372 loci as implemented in *Arlequin 2000 *[25]. AMOVA enables the partition of genetic variance at a locus or several loci into variation *within *populations and variation *between *populations. In addition, AMOVA can be used for a hierarchical analysis of three genetic-variance components – those due to genetic differences (i) between individuals within groups, (ii) between populations within groups, and (iii) between groups. We conducted AMOVA analyses on the study sample using two models (a) a model in that partitioned the genetic variance into that within each ethnic group and that between ethnic groups, (b) a hierarchical model with the country as the first level and the ethnic group within each country as the second level. Additional locus-by-locus AMOVA analysis was done (see Additional file 2). Significance of the AMOVA values was estimated by used of 10,000 permutations. F_ST_, the fixation index or coancestry coefficient [26], was also computed as a measure of the effect of population division. F_ST _ranges from 0 (no population subdivision, random mating occurrence, no genetic divergence within the population) to 1 (complete isolation or extreme division), and F_ST _values of up to 0.05 represents negligible genetic differentiation. Allele-sharing genetic distances [14] were also computed between each pair of ethnic groups.

## Authors' contributions

AA and CR conceived and designed the study; AA did the statistical genetic analyses; AA and CR drafted the manuscript. GC and YC contributed to the interpretation of the results and development of the manuscript.

## Supplementary Material

Additional File 1 The 372 microsatellite markers on the 22 autosomes studied Click here for file

Additional File 2 Single locus AMOVA for all 372 loci studied Click here for file
