# Supplementary material for: Genetic structure in four West African population groups
Source: BMC Genet. 2005 Jun 24;6:38. doi: 10.1186/1471-2156-6-38 (PMC1180433; doi:10.1186/1471-2156-6-38)
Supplement: Additional File 1 — The 372 microsatellite markers on the 22 autosomes studied [file 1471-2156-6-38-S1.pdf]

**Africa-America Diabetes Mellitus (AADM) Study**
**List of the 372 microsatellite markers on the 22 autosomes used in genetic structure analyses**

| Locus number | Locus name | Type [2=dinucleotide, 3=trinucleotide, 4=tetranucleotide] |
|---|---|---|
| 1 | D1S2845 | 2 |
| 2 | D1S2660 | 2 |
| 3 | D1S1612 | 4 |
| 4 | D1S1597 | 4 |
| 5 | D1S3669 | 4 |
| 6 | D1S552 | 4 |
| 7 | D1S1622 | 3 |
| 8 | D1S255 | 2 |
| 9 | D1S3721 | 4 |
| 10 | D1S2134 | 4 |
| 11 | D1S3728 | 4 |
| 12 | D1S1665 | 4 |
| 13 | D1S1728 | 4 |
| 14 | D1S551 | 4 |
| 15 | D1S1588 | 3 |
| 16 | D1S1631 | 3 |
| 17 | D1S3723 | 4 |
| 18 | D1S534 | 4 |
| 19 | D1S1653 | 4 |
| 20 | D1S1679 | 4 |
| 21 | D1S1677 | 4 |
| 22 | D1S1619 | 3 |
| 23 | D1S1589 | 3 |
| 24 | D1S518 | 4 |
| 25 | D1S1660 | 4 |
| 26 | D1S1647 | 4 |
| 27 | C1S1248 | 4 |
| 28 | D1S2141 | 4 |
| 29 | D1S549 | 4 |
| 30 | D1S3462 | 3 |
| 31 | D1S235 | 2 |
| 32 | D1S547 | 4 |
| 33 | D1S1609 | 4 |
| 34 | D2S2976 | 4 |
| 35 | D2S2952 | 4 |
| 36 | D2S1400 | 4 |
| 37 | D2S1360 | 4 |
| 38 | D2S405 | 4 |
| 39 | D2S1788 | 4 |
| 40 | D2S1356 | 3 |
| 41 | D2S2739 | 4 |
| 42 | D2S441 | 4 |
| 43 | D2S1394 | 4 |
| 44 | D2S1777 | 4 |
| 45 | D2S1790 | 4 |
| 46 | D2S2972 | 4 |
| 47 | D2S410 | 4 |
| 48 | D2S1328 | 4 |
| 49 | D2S1334 | 4 |
| 50 | D2S442 | 4 |
| 51 | D2S1399 | 4 |
| 52 | D2S1353 | 3 |
| 53 | D2S1776 | 4 |
| 54 | D2S1391 | 4 |
| 55 | D2S1384 | 4 |
| 56 | D2S2944 | 4 |

| | | |
|---|---|---|
| 57 | D2S434 | 4 |
| 58 | D2S1363 | 4 |
| 59 | D2S427 | 4 |
| 60 | D2S2968 | 4 |
| 61 | D2S125 | 2 |
| 62 | D3S2387 | 4 |
| 63 | D3S1560 | 2 |
| 64 | D3S1304 | 2 |
| 65 | D3S4545 | 4 |
| 66 | D3S1259 | 2 |
| 67 | D3S3038 | 4 |
| 68 | D3S2432 | 4 |
| 69 | D3S1768 | 4 |
| 70 | D3S2409 | 3 |
| 71 | D3S1766 | 4 |
| 72 | D3S4542 | 4 |
| 73 | D3S2406 | 4 |
| 74 | D3S4529 | 4 |
| 75 | D3S2459 | 4 |
| 76 | D3S3045 | 4 |
| 77 | D3S2460 | 4 |
| 78 | D3S4523 | 3 |
| 79 | D3S1764 | 4 |
| 80 | D3S1744 | 4 |
| 81 | D3S1763 | 4 |
| 82 | D3S3053 | 4 |
| 83 | D3S2427 | 4 |
| 84 | D3S1262 | 2 |
| 85 | D3S2398 | 4 |
| 86 | D3S2418 | 3 |
| 87 | D3S1311 | 2 |
| 88 | D4S2366 | 4 |
| 89 | D4S403 | 2 |
| 90 | D4S2639 | 4 |
| 91 | D4S2397 | 3 |
| 92 | D4S2632 | 4 |
| 93 | D4S1627 | 4 |
| 94 | D4S3248 | 4 |
| 95 | D4S2367 | 4 |
| 96 | D4S3243 | 4 |
| 97 | D4S2361 | 3 |
| 98 | D4S1647 | 4 |
| 99 | D4S2623 | 4 |
| 100 | D4S2394 | 3 |
| 101 | D4S1644 | 4 |
| 102 | D4S1625 | 4 |
| 103 | D4S1629 | 4 |
| 104 | D4S2368 | 4 |
| 105 | D4S2431 | 4 |
| 106 | D4S2417 | 4 |
| 107 | D4S408 | 2 |
| 108 | D4S1652 | 4 |
| 109 | D5S2488 | 3 |
| 110 | D5S2849 | 4 |
| 111 | D5S2505 | 4 |
| 112 | D5S807 | 4 |
| 113 | D5S817 | 4 |
| 114 | D5S2845 | 4 |
| 115 | D5S2848 | 4 |
| 116 | D5S1470 | 4 |
| 117 | D5S1457 | 4 |

| | | |
|---|---|---|
| 118 | D5S2500 | 4 |
| 119 | D5S424 | 2 |
| 120 | D5S641 | 2 |
| 121 | D5S1725 | 4 |
| 122 | D5S1503 | 4 |
| 123 | D5S1453 | 3 |
| 124 | D5S2501 | 4 |
| 125 | D5S1505 | 4 |
| 126 | D5S816 | 4 |
| 127 | D5S1480 | 3 |
| 128 | D5S820 | 4 |
| 129 | D5S1471 | 4 |
| 130 | D5S1456 | 4 |
| 131 | D5S211 | 2 |
| 132 | D5S408 | 2 |
| 133 | F13A1 | 4 |
| 134 | D6S2434 | 3 |
| 135 | D6S1959 | 4 |
| 136 | D6S2439 | 4 |
| 137 | D6S2427 | 4 |
| 138 | D6S1017 | 4 |
| 139 | D6S2410 | 4 |
| 140 | D6S1053 | 4 |
| 141 | D6S1031 | 3 |
| 142 | D6S1056 | 4 |
| 143 | D6S1021 | 3 |
| 144 | D6S474 | 4 |
| 145 | D6S1040 | 4 |
| 146 | D6S1009 | 4 |
| 147 | C6S1848 | 4 |
| 148 | D6S2436 | 4 |
| 149 | D6S1035 | 3 |
| 150 | D6S1277 | 4 |
| 151 | D6S1027 | 3 |
| 152 | D7S3056 | 4 |
| 153 | D7S513 | 2 |
| 154 | D7S3051 | 4 |
| 155 | D7S1802 | 4 |
| 156 | D7S1808 | 4 |
| 157 | D7S817 | 4 |
| 158 | D7S2846 | 4 |
| 159 | D7S1818 | 4 |
| 160 | D7S3046 | 4 |
| 161 | D7S2204 | 4 |
| 162 | D7S2212 | 4 |
| 163 | D7S821 | 4 |
| 164 | D7S1799 | 4 |
| 165 | D7S3061 | 4 |
| 166 | D7S1804 | 4 |
| 167 | D7S1824 | 4 |
| 168 | D7S2195 | 4 |
| 169 | D7S3070 | 4 |
| 170 | D7S3058 | 4 |
| 171 | D7S559 | 2 |
| 172 | D8S264 | 2 |
| 173 | D8S1469 | 4 |
| 174 | D8S1130 | 4 |
| 175 | D8S1106 | 4 |
| 176 | D8S1145 | 4 |
| 177 | D8S136 | 2 |
| 178 | D8S1771 | 2 |

| | | |
|---|---|---|
| 179 | D8S1477 | 4 |
| 180 | D8S1110 | 4 |
| 181 | D8S1113 | 4 |
| 182 | D8S1136 | 4 |
| 183 | D8S2324 | 4 |
| 184 | D8S1119 | 3 |
| 185 | C8S14 | 4 |
| 186 | D8S1132 | 4 |
| 187 | D8S592 | 4 |
| 188 | D8S1179 | 4 |
| 189 | D8S1128 | 4 |
| 190 | D8S256 | 2 |
| 191 | D8S373 | 4 |
| 192 | D9S2169 | 4 |
| 193 | D9S168 | 2 |
| 194 | D9S925 | 4 |
| 195 | D9S1121 | 4 |
| 196 | D9S1118 | 4 |
| 197 | D9S301 | 4 |
| 198 | D9S1122 | 4 |
| 199 | D9S922 | 4 |
| 200 | D9S283 | 2 |
| 201 | D9S1786 | 2 |
| 202 | D9S938 | 4 |
| 203 | D9S930 | 4 |
| 204 | D9S934 | 4 |
| 205 | D9S1825 | 2 |
| 206 | D9S2157 | 3 |
| 207 | D9S1826 | 2 |
| 208 | D9S1838 | 2 |
| 209 | D10S1435 | 4 |
| 210 | D10S189 | 2 |
| 211 | D10S1412 | 3 |
| 212 | D10S2325 | 4 |
| 213 | D10S1423 | 4 |
| 214 | D10S1426 | 4 |
| 215 | D10S1208 | 3 |
| 216 | D10S1221 | 3 |
| 217 | D10S1225 | 3 |
| 218 | C10S1218 | 4 |
| 219 | D10S1432 | 4 |
| 220 | D10S2327 | 4 |
| 221 | D10S2470 | 4 |
| 222 | D10S677 | 4 |
| 223 | D10S1239 | 4 |
| 224 | D10S1237 | 4 |
| 225 | D10S1230 | 3 |
| 226 | D10S1656 | 2 |
| 227 | D10S217 | 2 |
| 228 | D10S212 | 2 |
| 229 | D11S1984 | 4 |
| 230 | D11S2362 | 3 |
| 231 | D11S1999 | 4 |
| 232 | D11S1981 | 4 |
| 233 | C10S348 | 3 |
| 234 | D11S1392 | 4 |
| 235 | D11S1344 | 2 |
| 236 | D11S2371 | 4 |
| 237 | D11S2002 | 4 |
| 238 | D11S2000 | 4 |
| 239 | D11S1391 | 4 |

| | | |
|---|---|---|
| 240 | D11S1998 | 4 |
| 241 | D11S4464 | 4 |
| 242 | D11S912 | 2 |
| 243 | D11S968 | 2 |
| 244 | D12S372 | 4 |
| 245 | C12S4912 | 4 |
| 246 | D12S391 | 4 |
| 247 | D12S373 | 4 |
| 248 | D12S1042 | 4 |
| 249 | C12S916 | 4 |
| 250 | D12S398 | 4 |
| 251 | D12S1294 | 4 |
| 252 | D12S375 | 4 |
| 253 | D12S1052 | 4 |
| 254 | D12S1064 | 4 |
| 255 | D12S1300 | 4 |
| 256 | PAH | 4 |
| 257 | D12S2070 | 4 |
| 258 | D12S395 | 4 |
| 259 | D12S2078 | 4 |
| 260 | D12S1045 | 4 |
| 261 | D12S392 | 4 |
| 262 | D13S787 | 4 |
| 263 | D13S217 | 2 |
| 264 | D13S1493 | 4 |
| 265 | D13S894 | 4 |
| 266 | D13S325 | 4 |
| 267 | D13S788 | 4 |
| 268 | D13S800 | 4 |
| 269 | D13S317 | 4 |
| 270 | D13S793 | 4 |
| 271 | D13S779 | 3 |
| 272 | D13S796 | 4 |
| 273 | D13S1265 | 2 |
| 274 | D13S285 | 2 |
| 275 | D14S742 | 4 |
| 276 | D14S1280 | 4 |
| 277 | D14S608 | 4 |
| 278 | D14S599 | 3 |
| 279 | D14S306 | 4 |
| 280 | D14S587 | 4 |
| 281 | D14S592 | 3 |
| 282 | D14S588 | 4 |
| 283 | D14S53 | 2 |
| 284 | D14S606 | 4 |
| 285 | C14S1937 | 4 |
| 286 | D14S617 | 4 |
| 287 | D14S1434 | 4 |
| 288 | D14S1426 | 4 |
| 289 | D15S822 | 4 |
| 290 | D15S165 | 2 |
| 291 | D15S1012 | 2 |
| 292 | D15S659 | 4 |
| 293 | D15S643 | 4 |
| 294 | D15S1507 | 4 |
| 295 | D15S131 | 2 |
| 296 | D15S655 | 3 |
| 297 | D15S652 | 3 |
| 298 | D15S816 | 4 |
| 299 | D15S657 | 4 |
| 300 | D15S966 | 2 |

| | | |
|---|---|---|
| 301 | D15S642 | 4 |
| 302 | D16S2616 | 3 |
| 303 | D16S748 | 3 |
| 304 | D16S764 | 4 |
| 305 | D16S403 | 2 |
| 306 | D16S769 | 4 |
| 307 | D16S540 | 4 |
| 308 | D16S3396 | 3 |
| 309 | D16S3253 | 4 |
| 310 | D16S2624 | 4 |
| 311 | D16S3091 | 2 |
| 312 | D16S539 | 4 |
| 313 | D16S2621 | 4 |
| 314 | D17S1308 | 4 |
| 315 | D17S1298 | 4 |
| 316 | D17S974 | 4 |
| 317 | D17S1303 | 4 |
| 318 | D17S799 | 2 |
| 319 | D17S2196 | 4 |
| 320 | D17S975 | 4 |
| 321 | D17S1293 | 4 |
| 322 | D17S1299 | 4 |
| 323 | D17S2180 | 3 |
| 324 | D17S1290 | 4 |
| 325 | D17S2193 | 3 |
| 326 | D17S1301 | 4 |
| 327 | D17S784 | 2 |
| 328 | D17S928 | 2 |
| 329 | C18S1781 | 4 |
| 330 | D18S976 | 4 |
| 331 | D18S843 | 3 |
| 332 | D18S542 | 4 |
| 333 | D18S877 | 4 |
| 334 | D18S535 | 4 |
| 335 | D18S851 | 4 |
| 336 | D18S858 | 3 |
| 337 | D18S862 | 3 |
| 338 | D18S1364 | 4 |
| 339 | C18S822 | 3 |
| 340 | D18S1371 | 4 |
| 341 | D19S591 | 4 |
| 342 | D19S1034 | 4 |
| 343 | D19S586 | 4 |
| 344 | D19S714 | 4 |
| 345 | D19S433 | 4 |
| 346 | D19S245 | 2 |
| 347 | D19S178 | 4 |
| 348 | D19S246 | 4 |
| 349 | D19S589 | 4 |
| 350 | D19S254 | 4 |
| 351 | D20S103 | 2 |
| 352 | D20S482 | 4 |
| 353 | D20S851 | 2 |
| 354 | D20S604 | 4 |
| 355 | D20S470 | 4 |
| 356 | D20S477 | 4 |
| 357 | D20S478 | 4 |
| 358 | D20S481 | 4 |
| 359 | D20S480 | 4 |
| 360 | D20S171 | 2 |
| 361 | D21S1432 | 4 |

| | | |
|---|---|---|
| 362 | D21S1437 | 4 |
| 363 | D21S2052 | 4 |
| 364 | D21S1440 | 3 |
| 365 | D21S2055 | 4 |
| 366 | D21S1446 | 4 |
| 367 | D22S420 | 2 |
| 368 | D22S345 | 2 |
| 369 | D22S689 | 4 |
| 370 | D22S685 | 4 |
| 371 | D22S683 | 4 |
| 372 | D22S445 | 4 |
