# Supplementary material for: Genetic structure in four West African population groups
Source: BMC Genet. 2005 Jun 24;6:38. doi: 10.1186/1471-2156-6-38 (PMC1180433; doi:10.1186/1471-2156-6-38)
Supplement: Additional File 2 — Single locus AMOVA for all 372 loci studied [file 1471-2156-6-38-S2.pdf]

Africa-America Diabetes Mellitus (ADM) Study

****
Locus by locus AMOVA:
****

	Among Groups:				Among Population:				Within Populations:				Fixation indices:					
Locus	SSD	d.f.	Va	% variation	SSD	d.f.	Vb	% variation	SSD	d.f.	Vc	% variation	FSC	P-value	FST	P-value	FCT	P-value
1	1.41973	1	0.00230	0.51148	0.76699	2	-0.00030	-0.06634	414.79930	926	0.44795	99.55486	-0.00067	0.63436	0.00445	0.02059	0.00511	0.32950
2	0.39669	1	0.00040	0.10799	0.45933	2	-0.00063	-0.17089	354.91634	958	0.37048	100.06290	-0.00171	0.72871	-0.00063	0.64505	0.00108	0.33446
3	1.63732	1	0.00268	0.62097	0.93002	2	0.00017	0.04028	376.17356	876	0.42942	99.33876	0.00041	0.36297	0.00661	0.00475	0.00621	0.34040
4	0.87489	1	0.00100	0.24873	0.80540	2	0.00001	0.00129	387.08624	964	0.40154	99.74998	0.00001	0.39109	0.00250	0.11673	0.00249	0.32970
5	0.24634	1	0.00005	0.01071	0.50676	2	-0.00089	-0.20567	375.50263	868	0.43261	100.19495	-0.00206	0.88109	-0.00195	0.94079	0.00011	0.66941
6	0.07175	1	-0.00107	-0.30157	1.09126	2	0.00086	0.24144	340.31205	958	0.35523	100.06013	0.00241	0.18495	-0.00060	0.38574	-0.00302	1.00000
7	1.95305	1	0.00329	0.84120	0.87197	2	0.00023	0.05765	363.27583	938	0.38729	99.10115	0.00058	0.32139	0.00899	0.00426	0.00841	0.33188
8	0.54016	1	0.00045	0.11485	0.66985	2	-0.00027	-0.06846	379.71323	960	0.39553	99.95361	-0.00069	0.58663	0.00046	0.42624	0.00115	0.33614
9	0.48218	1	-0.00000	-0.00032	0.94362	2	0.00031	0.07666	392.54155	976	0.40219	99.92366	0.00077	0.31020	0.00076	0.29208	-0.00000	0.65396
10	0.69197	1	0.00046	0.12172	0.93877	2	0.00042	0.11088	350.26474	924	0.37907	99.76740	0.00111	0.25455	0.00233	0.14149	0.00122	0.33772
11	0.87106	1	0.00028	0.06594	1.38882	2	0.00120	0.27972	411.39747	962	0.42765	99.65434	0.00280	0.07287	0.00346	0.02218	0.00066	0.66950
12	0.79073	1	0.00092	0.21646	0.73529	2	-0.00025	-0.05935	405.95215	958	0.42375	99.84290	-0.00059	0.55683	0.00157	0.23376	0.00216	0.34228
13	1.12010	1	0.00187	0.56558	0.51149	2	-0.00034	-0.10190	314.01172	952	0.32984	99.53632	-0.00102	0.59614	0.00464	0.08426	0.00566	0.33050
14	0.36458	1	0.00074	0.21028	0.11604	2	-0.00132	-0.37491	336.79542	956	0.35230	100.16463	-0.00376	0.98901	-0.00165	0.89772	0.00210	0.33386
15	1.77568	1	0.00309	1.00002	0.66095	2	0.00011	0.03548	290.94911	950	0.30626	98.96450	0.00036	0.32901	0.01036	0.01475	0.01000	0.34436
16	0.62321	1	0.00008	0.01732	1.13381	2	0.00061	0.14128	406.99458	938	0.43390	99.84140	0.00141	0.22356	0.00159	0.15792	0.00017	0.33307
17	0.61434	1	-0.00016	-0.03739	1.30700	2	0.00106	0.25073	399.86109	952	0.42002	99.78666	0.00251	0.10198	0.00213	0.08792	-0.00037	0.66941
18	1.35947	1	0.00112	0.26586	1.58044	2	0.00171	0.40449	392.50477	936	0.41934	99.32964	0.00406	0.03436	0.00670	0.00218	0.00266	0.32644
19	0.08361	1	-0.00036	-0.11266	0.52696	2	-0.00028	-0.08561	308.86746	952	0.32444	100.19827	-0.00086	0.54495	-0.00198	0.75287	-0.00113	1.00000
20	0.34826	1	-0.00007	-0.01710	0.77132	2	-0.00013	-0.03167	401.16909	966	0.41529	100.04877	-0.00032	0.49842	-0.00049	0.55891	-0.00017	1.00000
21	0.96113	1	0.00112	0.28886	0.87451	2	0.00024	0.06196	362.63054	942	0.38496	99.64918	0.00062	0.31574	0.00351	0.08505	0.00289	0.32752
22	0.38778	1	0.00002	0.00549	0.74862	2	0.00012	0.03578	329.15876	948	0.34721	99.95873	0.00036	0.37198	0.00041	0.37109	0.00005	0.66515
23	1.29109	1	0.00225	0.54679	0.50687	2	-0.00071	-0.17122	394.03917	960	0.41046	99.62443	-0.00172	0.79059	0.00376	0.11356	0.00547	0.32871
24	0.57269	1	0.00064	0.15113	0.59436	2	-0.00059	-0.13851	400.55705	942	0.42522	99.98738	-0.00139	0.77693	0.00013	0.56119	0.00151	0.33505
25	0.19264	1	-0.00030	-0.07628	0.68166	2	-0.00025	-0.06320	372.27475	942	0.39520	100.13948	-0.00063	0.52396	-0.00139	0.71426	-0.00076	1.00000
26	0.59274	1	-0.00040	-0.09103	1.46953	2	0.00138	0.31195	398.28618	900	0.44254	99.77908	0.00312	0.04703	0.00221	0.03950	-0.00091	1.00000
27	0.02337	1	-0.00049	-0.14750	0.51479	2	-0.00037	-0.10965	308.08955	920	0.33488	100.25715	-0.00109	0.57535	-0.00257	0.82030	-0.00148	1.00000
28	0.71431	1	0.00021	0.05141	1.18216	2	0.00080	0.19345	393.37759	952	0.41321	99.75514	0.00194	0.16851	0.00245	0.09198	0.00051	0.66396
29	0.95741	1	0.00077	0.19326	1.13967	2	0.00077	0.19409	380.90812	958	0.39761	99.61265	0.00194	0.17317	0.00387	0.05663	0.00193	0.32812
30	0.26926	1	-0.00017	-0.04448	0.71062	2	-0.00015	-0.03819	371.15658	956	0.38824	100.08267	-0.00038	0.49713	-0.00083	0.62376	-0.00044	0.66416
31	0.33390	1	-0.00024	-0.06558	0.87409	2	0.00029	0.07847	354.31507	950	0.37296	99.98712	0.00078	0.27505	0.00013	0.33485	-0.00066	1.00000
32	0.18843	1	-0.00164	-0.47505	1.72730	2	0.00239	0.69302	322.10661	936	0.34413	99.78203	0.00690	0.03574	0.00218	0.07990	-0.00475	0.66554
33	0.13363	1	-0.00059	-0.13637	0.81998	2	-0.00010	-0.02375	411.09775	950	0.43273	100.16012	-0.00024	0.49158	-0.00160	0.79950	-0.00136	1.00000
34	0.86981	1	0.00154	0.38555	0.41985	2	-0.00089	-0.22265	363.96907	912	0.39909	99.83710	-0.00224	0.94475	0.00163	0.47050	0.00386	0.33069
35	0.39109	1	-0.00101	-0.23502	1.61785	2	0.00171	0.39696	412.05433	960	0.42922	99.83806	0.00396	0.02881	0.00162	0.05832	-0.00235	1.00000
36	0.58445	1	0.00069	0.16101	0.56479	2	-0.00067	-0.15527	409.83402	952	0.43050	99.99426	-0.00156	0.83168	0.00006	0.62228	0.00161	0.32832
37	0.66083	1	0.00113	0.27416	0.33118	2	-0.00111	-0.26967	395.96017	958	0.41332	99.99551	-0.00270	0.96020	0.00004	0.68109	0.00274	0.33188
38	0.12478	1	-0.00116	-0.30570	1.27673	2	0.00115	0.30168	367.87890	966	0.38083	100.00402	0.00301	0.09960	-0.00004	0.24604	-0.00306	1.00000
39	1.61204	1	0.00255	0.58634	0.90273	2	0.00009	0.02056	398.60294	922	0.43232	99.39310	0.00021	0.42851	0.00607	0.01178	0.00586	0.32564
40	0.32621	1	-0.00054	-0.13118	1.11181	2	0.00066	0.16154	392.64704	960	0.40901	99.96964	0.00161	0.18139	0.00030	0.28772	-0.00131	0.66851
41	0.61344	1	0.00031	0.06568	0.94200	2	-0.00003	-0.00714	443.91666	928	0.47836	99.94146	-0.00007	0.50861	0.00059	0.29901	0.00066	0.33376
42	1.61389	1	0.00256	0.67894	0.88628	2	0.00032	0.08579	344.86592	922	0.37404	99.23527	0.00086	0.27861	0.00765	0.01178	0.00679	0.33228
43	0.41247	1	0.00037	0.09647	0.51817	2	-0.00055	-0.14473	362.86748	952	0.38116	100.04826	-0.00145	0.69465	-0.00048	0.62842	0.00096	0.32337
44	0.25989	1	-0.00029	-0.07369	0.79692	2	0.00000	0.00093	382.52808	962	0.39764	100.07276	0.00001	0.45683	-0.00073	0.59901	-0.00074	0.65792
45	0.56934	1	0.00008	0.01927	1.03578	2	0.00042	0.09981	399.55908	940	0.42506	99.88092	0.00100	0.26426	0.00119	0.20752	0.00019	0.65990
46	0.86025	1	0.00132	0.34227	0.51697	2	-0.00057	-0.14702	369.23089	958	0.38542	99.80475	-0.00148	0.72475	0.00195	0.24277	0.00342	0.32396
47	0.96036	1	-0.00061	-0.13988	2.29450	2	0.00316	0.71963	422.82950	968	0.43681	99.42025	0.00719	0.00168	0.00580	0.00020	-0.00140	0.66248
48	0.30265	1	-0.00043	-0.10796	0.97660	2	0.00040	0.09918	373.83424	930	0.40197	100.00879	0.00099	0.26069	-0.00009	0.35495	-0.00108	0.66515
49	0.29875	1	-0.00030	-0.06724	0.88779	2	-0.00003	-0.00695	437.39867	970	0.45093	100.07419	-0.00007	0.51911	-0.00074	0.69782	-0.00067	0.66485
50	0.18397	1	-0.00082	-0.20287	1.08684	2	0.00063	0.15442	381.61241	938	0.40684	100.04845	0.00154	0.18703	-0.00048	0.37356	-0.00203	1.00000
51	0.40126	1	-0.00008	-0.01899	0.87570	2	0.00005	0.01268	413.77632	972	0.42570	100.00631	0.00013	0.42000	-0.00006	0.45861	-0.00019	0.66782
52	0.63980	1	0.00041	0.10735	0.88474	2	0.00029	0.07646	354.77503	934	0.37984	99.81619	0.00077	0.28683	0.00184	0.16871	0.00107	0.66861
53	0.54437	1	-0.00006	-0.01551	1.09045	2	0.00077	0.20637	362.66008	976	0.37158	99.80914	0.00206	0.17030	0.00191	0.12168	-0.00016	0.66554
54	0.35186	1	0.00040	0.11065	0.39290	2	-0.00074	-0.20827	336.93072	942	0.35768	100.09762	-0.00209	0.78614	-0.00098	0.73030	0.00111	0.33683
55	0.92981	1	0.00144	0.40061	0.52789	2	-0.00042	-0.11730	345.89457	964	0.35881	99.71669	-0.00118	0.61832	0.00283	0.19287	0.00401	0.33257
56	0.12165	1	-0.00023	-0.05999	0.50330	2	-0.00062	-0.15967	374.99538	960	0.39062	100.21966	-0.00160	0.75505	-0.00220	0.90267	-0.00060	1.00000
57	0.86784	1	0.00101	0.25247	0.79428	2	-0.00000	-0.00014	377.39449	950	0.39726	99.74766	-0.00000	0.41891	0.00252	0.15990	0.00252	0.32931
58	1.57403	1	0.00259	0.66066	0.76050	2	-0.00004	-0.01131	366.50340	940	0.38990	99.35065	-0.00011	0.45188	0.00649	0.02406	0.00661	0.33832
59	0.75502	1	0.00093	0.24061	0.66471	2	-0.00023	-0.06072	364.60019	950	0.38379	99.82011	-0.00061	0.53198	0.00180	0.24188	0.00241	0.32396
60	0.78217	1	0.00098	0.26263	0.66881	2	-0.00017	-0.04484	352.00700	948	0.37132	99.78222	-0.00045	0.48554	0.00218	0.21198	0.00263	0.33208
61	0.39370	1	0.00000	0.00120	0.78535	2	-0.00003	-0.00815	374.11137	936	0.39969	100.00695	-0.00008	0.46564	-0.00007	0.48099	0.00001	0.66772
62	0.96870	1	0.00157	0.34407	0.51613	2	-0.00089	-0.19477	439.78377	964	0.45621	99.85070	-0.00195	0.96475	0.00149	0.38851	0.00344	0.33406
63	0.63826	1	0.00053	0.12111	0.86227	2	-0.00002	-0.00545	350.12695	804	0.43548	99.88433	-0.00005	0.47624	0.00116	0.30485	0.00121	0.66505
64	0.83221	1	0.00046	0.11448	1.17815	2	0.00086	0.21267	379.47697	944	0.40199	99.67285	0.00213	0.13822	0.00327	0.04812	0.00114	0.33653
65	0.47031	1	-0.00015	-0.03492	1.05542	2	0.00041	0.09346	414.25006	946	0.43790	99.94145	0.00093	0.23149	0.00059	0.24475	-0.00035	0.66832
66	0.40009	1	-0.00079	-0.18692	1.43829	2	0.00136	0.32384	397.29453	944	0.42086	99.86309	0.00323	0.04851	0.00137	0.07842	-0.00187	0.66030
67	0.22530	1	-0.00034	-0.08214	0.77157	2	-0.00011	-0.02685	390.31276	952	0.40999	100.10899	-0.00027	0.48149	-0.00109	0.66693	-0.00082	1.00000
68	0.49341	1	0.00052	0.12399	0.55782	2	-0.00064	-0.15454	383.84595	920	0.41722	100.03055	-0.00155	0.76762	-0.00031	0.62099	0.00124	0.33574
69	0.69686	1	0.00086	0.21177	0.60446	2	-0.00047	-0.11485	394.53819	968	0.40758	99.90308	-0.00115	0.64851	0.00097	0.33089	0.00212	0.32663
70	1.21923	1	0.00177	0.47311	0.94033	2	0.00052	0.13917	307.95675	830	0.37103	99.38772	0.00140	0.22584	0.00612	0.02990	0.00473	0.33119
71	0.26294	1	-0.00051	-0.13840	0.95475	2	0.00050	0.13457	343.93392	926	0.37142	100.00383	0.00134	0.28099	-0.00004	0.38941	-0.00138	0.67287
72	1.19589	1	0.00127	0.33078	1.17371	2	0.00094	0.24535	355.99871	930	0.38279	99.42387	0.00246	0.21535	0.00576	0.04960	0.00331	0.33614
73	0.68985	1	-0.00014	-0.03098	1.42348	2	0.00128	0.28606	394.18192	880	0.44793	99.74491	0.00286	0.05069	0.00255	0.03248	-0.00031	0.33604
74	0.35100	1	0.00055	0.14781	0.26136	2	-0.00111	-0.29946	341.29219	918	0.37178	100.15164	-0.00300	0.93604	-0.00152	0.86535	0.00148	0.33960
75	0.24387	1	-0.00035	-0.08217	0.81624	2	-0.00006	-0.01400	403.50434	958	0.42119	100.09617	-0.00014	0.44307	-0.00096	0.62257	-0.00082	1.00000
76	0.77665	1	0.00023	0.05859	1.26193	2	0.00108	0.26906	371.65414	930	0.39963	99.67234	0.00269	0.10168	0.00328	0.04614	0.00059	0.66505
77	0.25298	1	-0.00255	-0.70452	2.47033	2	0.00425	1.17644	317.33989	882	0.35980	99.52808	0.01168	0.00455	0.00472	0.01050	-0.00705	1.00000

78	1.23616	1	0.00153	0.39194	1.00328	2	0.00051	0.13040	370.01528	950	0.38949	99.47767	0.00131	0.19545	0.00522	0.01812	0.00392	0.33832
79	1.36103	1	0.00092	0.24996	1.71348	2	0.00222	0.60311	348.68123	954	0.36549	99.14693	0.00605	0.01545	0.00853	0.00109	0.00250	0.66178
80	0.55246	1	0.00096	0.24680	0.29143	2	−0.00112	−0.28710	369.04137	946	0.39011	100.04030	−0.00288	0.95475	−0.00040	0.73812	0.00247	0.33723
81	0.70894	1	0.00129	0.32491	0.35094	2	−0.00107	−0.26996	355.53478	894	0.39769	99.94505	−0.00271	0.91277	0.00055	0.56703	0.00325	0.33426
82	0.29331	1	−0.00035	−0.09985	0.88808	2	0.00042	0.12017	336.84966	962	0.35016	99.97969	0.00120	0.25386	0.00020	0.32743	−0.00100	0.66188
83	0.87122	1	0.00105	0.22308	0.87314	2	−0.00016	−0.03499	401.99693	858	0.46853	99.81192	−0.00035	0.55000	0.00188	0.11426	0.00223	0.33683
84	0.46959	1	−0.00046	−0.10818	1.30765	2	0.00102	0.24070	412.35773	974	0.42337	99.86747	0.00240	0.09574	0.00133	0.12119	−0.00108	1.00000
85	1.10544	1	0.00182	0.48452	0.54182	2	−0.00047	−0.12550	355.93136	950	0.37466	99.64098	−0.00126	0.63901	0.00359	0.14842	0.00485	0.34020
86	0.54686	1	0.00018	0.04568	0.90395	2	0.00026	0.06517	381.84445	968	0.39447	99.88915	0.00065	0.29653	0.00111	0.22713	0.00046	0.66099
87	0.90205	1	−0.00019	−0.04488	1.84315	2	0.00220	0.51672	411.81229	970	0.42455	99.52816	0.00516	0.00911	0.00472	0.00238	−0.00045	0.65911
88	0.39166	1	−0.00196	−0.53409	2.33865	2	0.00372	1.01031	341.25046	932	0.36615	99.52378	0.01005	0.00406	0.00476	0.00624	−0.00534	1.00000
89	0.24714	1	−0.00210	−0.50830	2.21122	2	0.00319	0.77214	389.73007	946	0.41198	99.73617	0.00768	0.00287	0.00264	0.01198	−0.00508	1.00000
90	0.22161	1	−0.00018	−0.04459	0.62662	2	−0.00039	−0.09845	370.08646	930	0.39794	100.14304	−0.00098	0.63178	−0.00143	0.76010	−0.00045	0.66950
91	1.11184	1	0.00097	0.24285	1.24582	2	0.00104	0.26083	374.84276	944	0.39708	99.49632	0.00261	0.10475	0.00504	0.01693	0.00243	0.33158
92	0.65944	1	−0.00025	−0.05801	1.47006	2	0.00139	0.31684	401.87375	920	0.43682	99.74117	0.00317	0.05248	0.00259	0.03495	−0.00058	0.66851
93	0.17115	1	0.00008	0.01974	0.33669	2	−0.00101	−0.25952	366.97739	944	0.38875	100.23978	−0.00260	0.90089	−0.00240	0.95337	0.00020	0.66792
94	0.32678	1	0.00020	0.05247	0.50620	2	−0.00057	−0.15008	361.18058	954	0.37860	100.09761	−0.00150	0.70228	−0.00098	0.69495	0.00052	0.33277
95	0.47989	1	0.00066	0.18795	0.39292	2	−0.00070	−0.19863	332.03055	948	0.35024	100.01068	−0.00199	0.76554	−0.00011	0.57376	0.00188	0.33109
96	0.46063	1	0.00017	0.05409	0.74311	2	0.00024	0.07549	306.84987	966	0.31765	99.87042	0.00076	0.31970	0.00130	0.26663	0.00054	0.66723
97	0.46819	1	−0.00112	−0.28127	1.83859	2	0.00234	0.58734	380.95829	958	0.39766	99.69393	0.00586	0.02149	0.00306	0.03149	−0.00281	1.00000
98	1.84068	1	0.00382	0.94212	0.21913	2	−0.00133	−0.32860	381.56309	948	0.40249	99.38648	−0.00332	0.98505	0.00614	0.04772	0.00942	0.33980
99	0.37356	1	0.00023	0.05305	0.57796	2	−0.00068	−0.15543	417.72735	952	0.43879	100.10238	−0.00156	0.83535	−0.00102	0.83624	0.00053	0.32842
100	1.47614	1	0.00154	0.38672	1.43310	2	0.00148	0.37125	370.92339	940	0.39460	99.24203	0.00373	0.07149	0.00758	0.00495	0.00387	0.32535
101	0.32501	1	0.00002	0.00501	0.65503	2	−0.00037	−0.08991	390.05858	954	0.40887	100.08490	−0.00090	0.62683	−0.00085	0.67624	0.00005	0.66703
102	0.56901	1	0.00061	0.16137	0.58567	2	−0.00037	−0.09893	364.74553	968	0.37680	99.93756	−0.00099	0.58644	0.00062	0.40881	0.00161	0.33327
103	0.52333	1	0.00061	0.23647	0.48099	2	−0.00007	−0.02817	245.27381	956	0.25656	99.79170	−0.00028	0.39653	0.00208	0.20931	0.00236	0.33713
104	0.13233	1	0.00006	0.01665	0.27938	2	−0.00098	−0.27622	338.48913	954	0.35481	100.25958	−0.00276	0.91079	−0.00260	0.96109	0.00017	0.67168
105	0.57022	1	0.00094	0.22866	0.36233	2	−0.00108	−0.26198	375.34959	914	0.41067	100.03333	−0.00263	0.96594	−0.00033	0.76703	0.00229	0.33089
106	0.25294	1	−0.00005	−0.01570	0.56759	2	−0.00022	−0.06655	311.28244	938	0.33186	100.08225	−0.00067	0.47158	−0.00082	0.53921	−0.00016	0.65990
107	1.04250	1	0.00038	0.09712	1.60502	2	0.00193	0.49900	360.95417	940	0.38399	99.40388	0.00499	0.03990	0.00596	0.01040	0.00097	0.33554
108	0.10419	1	−0.00070	−0.21287	0.84286	2	0.00042	0.12760	317.77368	970	0.32760	100.08527	0.00127	0.22673	−0.00085	0.40851	−0.00213	1.00000
109	0.96485	1	0.00178	0.46389	0.32812	2	−0.00098	−0.25603	368.09546	964	0.38184	99.79214	−0.00257	0.91515	0.00208	0.32010	0.00464	0.32792
110	0.62434	1	0.00053	0.13615	0.75950	2	−0.00006	−0.01445	366.86509	936	0.39195	99.87830	−0.00014	0.43673	0.00122	0.26891	0.00136	0.33327
111	0.32342	1	−0.00064	−0.15081	1.18818	2	0.00077	0.18197	398.80263	938	0.42516	99.96884	0.00182	0.18129	0.00031	0.28733	−0.00151	1.00000
112	0.35914	1	0.00005	0.01175	0.69569	2	−0.00031	−0.07495	395.15377	950	0.41595	100.06320	−0.00075	0.59653	−0.00063	0.61584	0.00012	0.67307
113	0.31010	1	−0.00062	−0.17435	1.12817	2	0.00096	0.27028	334.65139	944	0.35450	99.90407	0.00270	0.15129	0.00096	0.21495	−0.00174	1.00000
114	0.92435	1	0.00047	0.12284	1.33439	2	0.00132	0.34120	355.34255	924	0.38457	99.53596	0.00342	0.10584	0.00464	0.03832	0.00123	0.66881
115	0.12167	1	−0.00140	−0.34194	1.43001	2	0.00141	0.34443	379.09594	924	0.41028	99.99751	0.00343	0.07960	0.00002	0.22812	−0.00342	1.00000
116	0.53758	1	0.00054	0.13773	0.59019	2	−0.00042	−0.10712	380.30896	976	0.38966	99.96938	−0.00107	0.63762	0.00031	0.45733	0.00138	0.34446
117	1.04209	1	0.00208	0.55786	0.18191	2	−0.00125	−0.33520	362.95290	974	0.37264	99.77734	−0.00337	0.97861	0.00223	0.30030	0.00558	0.32713
118	0.14681	1	−0.00156	−0.36525	1.65506	2	0.00178	0.41737	415.32900	974	0.42642	99.94788	0.00416	0.02099	0.00052	0.11218	−0.00365	1.00000
119	0.14800	1	−0.00022	−0.05863	0.53513	2	−0.00046	−0.12556	357.51108	964	0.37086	100.18420	−0.00125	0.65485	−0.00184	0.83822	−0.00059	1.00000
120	0.61772	1	−0.00009	−0.01916	1.25163	2	0.00083	0.18587	419.75565	940	0.44655	99.83329	0.00186	0.13772	0.00167	0.09228	−0.00019	0.66653
121	0.62827	1	0.00097	0.26008	0.40858	2	−0.00076	−0.20447	355.29856	956	0.37165	99.94439	−0.00205	0.81129	0.00056	0.48614	0.00260	0.32584
122	0.85616	1	−0.00024	−0.06146	1.78099	2	0.00228	0.59202	368.57772	964	0.38234	99.46944	0.00592	0.02406	0.00531	0.01178	−0.00061	0.66277
123	0.27574	1	−0.00142	−0.41831	1.67283	2	0.00236	0.69315	308.38951	910	0.33889	99.72516	0.00690	0.02188	0.00275	0.04505	−0.00418	1.00000
124	0.66917	1	0.00010	0.02566	1.18307	2	0.00091	0.23210	369.88455	942	0.39266	99.74224	0.00232	0.15594	0.00258	0.10158	0.00026	0.66505
125	0.21625	1	−0.00068	−0.16330	1.03150	2	0.00045	0.10754	390.84799	936	0.41757	100.05577	0.00107	0.30109	−0.00056	0.50228	−0.00163	1.00000
126	0.68043	1	0.00029	0.07105	1.05471	2	0.00053	0.12836	387.92766	942	0.41181	99.80059	0.00128	0.24812	0.00199	0.14653	0.00071	0.67129
127	0.96908	1	0.00144	0.34811	0.65769	2	−0.00038	−0.09299	380.94957	926	0.41139	99.74488	−0.00093	0.59881	0.00255	0.15515	0.00348	0.33554
128	0.69028	1	0.00075	0.19430	0.68848	2	−0.00019	−0.04990	369.92437	956	0.38695	99.85560	−0.00050	0.55752	0.00144	0.32109	0.00194	0.33079
129	0.22447	1	−0.00081	−0.20759	1.16084	2	0.00085	0.21587	377.31200	964	0.39140	99.99172	0.00215	0.13574	0.00008	0.27327	−0.00208	1.00000
130	0.21176	1	0.00003	0.00841	0.44241	2	−0.00068	−0.18503	345.96154	938	0.36883	100.17662	−0.00185	0.76535	−0.00177	0.84703	0.00008	0.66822
131	0.91378	1	0.00067	0.16473	1.14556	2	0.00075	0.18355	389.03753	956	0.40694	99.65172	0.00184	0.15436	0.00348	0.04960	0.00165	0.32861
132	0.45998	1	−0.00128	−0.31156	1.95886	2	0.00256	0.62546	390.30616	956	0.40827	99.68609	0.00624	0.01198	0.00314	0.02208	−0.00312	0.66851
133	0.76936	1	0.00003	0.00776	1.41055	2	0.00138	0.34666	384.82112	972	0.39591	99.64558	0.00347	0.05059	0.00354	0.02485	0.00008	0.66020
134	1.88143	1	0.00392	0.97637	0.27117	2	−0.00121	−0.30048	370.17787	928	0.39890	99.32411	−0.00303	0.96970	0.00676	0.03525	0.00976	0.33772
135	0.15967	1	−0.00062	−0.18881	0.84733	2	0.00045	0.13545	299.31248	910	0.32891	100.05336	0.00135	0.40099	−0.00053	0.57584	−0.00189	1.00000
136	0.58909	1	0.00037	0.08909	0.82673	2	−0.00002	−0.00397	403.72204	968	0.41707	99.91488	−0.00004	0.47426	0.00085	0.35693	0.00089	0.66990
137	0.80101	1	0.00011	0.02561	1.42745	2	0.00129	0.29957	405.43469	946	0.42858	99.67482	0.00300	0.05505	0.00325	0.02139	0.00026	0.33446
138	1.64683	1	0.00277	0.70805	0.66563	2	−0.00025	−0.06296	378.13130	974	0.38823	99.35491	−0.00063	0.54752	0.00645	0.02693	0.00708	0.33119
139	0.10827	1	−0.00008	−0.02338	0.35252	2	−0.00084	−0.23129	347.65332	960	0.36214	100.25467	−0.00231	0.84307	−0.00255	0.94455	−0.00023	0.66901
140	0.33270	1	0.00016	0.04096	0.55092	2	−0.00052	−0.13455	360.82776	928	0.38882	100.09359	−0.00135	0.66396	−0.00094	0.67574	0.00041	0.65772
141	0.41641	1	−0.00016	−0.03920	0.95684	2	0.00036	0.09160	379.70280	956	0.39718	99.94759	0.00092	0.27515	0.00052	0.29168	−0.00039	1.00000
142	0.83277	1	0.00066	0.15871	1.02917	2	0.00046	0.11137	388.27423	936	0.41482	99.72992	0.00112	0.24881	0.00270	0.08663	0.00159	0.33663
143	1.38623	1	0.00256	0.61512	0.46804	2	−0.00083	−0.20038	389.21579	938	0.41494	99.58525	−0.00202	0.84762	0.00415	0.09465	0.00615	0.33129
144	0.30053	1	−0.00091	−0.23590	1.35981	2	0.00134	0.34590	365.27019	946	0.38612	99.89000	0.00345	0.10238	0.00110	0.18257	−0.00236	1.00000
145	0.49775	1	0.00060	0.18703	0.45514	2	−0.00041	−0.12760	309.52350	970	0.31910	99.94057	−0.00128	0.60911	0.00059	0.43149	0.00187	0.32525
146	0.80712	1	0.00095	0.22957	0.74286	2	−0.00018	−0.04448	391.26763	950	0.41186	99.81491	−0.00045	0.55693	0.00185	0.22673	0.00230	0.33436
147	1.58709	1	0.00157	0.36646	1.58964	2	0.00167	0.38825	406.57587	954	0.42618	99.24529	0.00390	0.03931	0.00755	0.00079	0.00366	0.32851
148	1.35533	1	0.00194	0.51745	0.86173	2	0.00026	0.07015	359.85907	968	0.37176	99.41240	0.00071	0.27158	0.00588	0.01861	0.00517	0.32812
149	1.27852	1	0.00023	0.06130	2.12702	2	0.00313	0.82131	357.36920	946	0.37777	99.11739	0.00822	0.01109	0.00883	0.00139	0.00061	0.66376
150	0.79831	1	0.00081	0.19261	0.86404	2	0.00006	0.01396	386.57307	922	0.41928	99.79342	0.00014	0.41158	0.00207	0.16594	0.00193	0.33376
151	0.63963	1	0.00079	0.18782	0.57127	2	−0.00061	−0.14475	407.75817	966	0.42211	99.95693	−0.00145	0.77762	0.00043	0.51287	0.00188	0.32832
152	0.49128	1	−0.00007	−0.01829	1.00273	2	0.00069	0.19238	313.01972	870	0.35979	99.82591	0.00192	0.20396	0.00174	0.18277	−0.00018	0.66931
153	0.46164	1	0.00025	0.05512	0.72431	2	−0.00042	−0.09199	423.94569	938	0.45197	100.03687	−0.00092	0.72921	−0.00037	0.67347	0.00055	0.65465
154	0.82047	1	0.00029	0.06973	1.29758	2	0.00106	0.25712	396.31362	962	0.41197	99.67314	0.00257	0.10010	0.00327	0.03634	0.00070	0.66634
155	1.03346	1	0.00076	0.20916	1.26732	2	0.00123	0.34051	346.39902	962	0.36008	99.45033	0.00341	0.07604	0.00550	0.01574	0.00209	0.66772
156	0.32589	1	−0.00028	−0.06851	0.90536	2	0.00017	0.04061	394.89034	950	0.41567	100.02790	0.00041	0.36099	−0.00028	0.47554	−0.00069	0.66990
157	0.43455	1	0.00039	0.09756	0.55209	2	−0.00059	−0.14673	365.60417	910	0.40176	100.04917	−0.00147	0.71842	−0.00049	0.63782	0.00098	0.66535
158	0.72426	1	0.00128	0.34625	0.30222	2	−0.00099	−0.26566	357.43530	964	0.37078	99.91941	−0.00267	0.89495	0.00081	0.48990	0.00346	0.33218
159	1.06755	1	0.00108	0.29648	1.09938	2	0.00087	0.23804	337.68914	934	0.36155	99.46548	0.00239	0.14564	0.00535	0.03307	0.00296	0.33386
160	0.54371	1	0.00013	0.03009	0.95192	2	0.00013	0.02923	429.05105	960	0.44693	99.94068	0.00029	0.37079	0.00059	0.30337	0.00030	0.33980
161	1.53193	1	0.00201	0.47097	1.11752	2	0.00060	0.14016	410.00622	966	0.42444	99.38888	0.00141	0.16990	0.00611	0.00545	0.00471	0.33822
162	0.25744	1	−0.00052	−0.14184	0.95391	2	0.00052	0.14118	339.49252	928	0.36583	100.00066	0.00141	0.25416	−0.00001	0.35832	−0.00142	1.00000

163	0.20760	1	-0.00099	-0.25930	1.24236	2	0.00112	0.29314	353.45240	928	0.38088	99.96616	0.00292	0.12030	0.00034	0.24990	-0.00259	0.67446
164	0.40128	1	0.00009	0.02447	0.72144	2	-0.00012	-0.03133	371.46460	958	0.38775	100.00686	-0.00031	0.46356	-0.00007	0.45218	0.00024	0.66168
165	0.42998	1	0.00034	0.08663	0.57061	2	-0.00051	-0.12756	381.34140	958	0.39806	100.04092	-0.00128	0.71465	-0.00041	0.62733	0.00087	0.66515
166	0.67932	1	0.00117	0.25658	0.35339	2	-0.00126	-0.27755	432.62557	950	0.45540	100.02097	-0.00278	0.99535	-0.00021	0.83297	0.00257	0.34386
167	0.69880	1	0.00090	0.21428	0.61133	2	-0.00052	-0.12418	389.68025	932	0.41811	99.90989	-0.00124	0.75158	0.00090	0.46109	0.00214	0.32663
168	0.71428	1	-0.00022	-0.04921	1.53700	2	0.00150	0.33194	411.64438	916	0.44939	99.71728	0.00332	0.03842	0.00283	0.02139	-0.00049	0.66059
169	0.37050	1	-0.00070	-0.16844	1.32931	2	0.00112	0.26902	398.17986	960	0.41477	99.89942	0.00269	0.09970	0.00101	0.14941	-0.00168	1.00000
170	0.31158	1	-0.00054	-0.12638	1.09055	2	0.00052	0.12159	408.40459	948	0.43081	100.00478	0.00121	0.22842	-0.00005	0.34832	-0.00126	1.00000
171	0.87946	1	0.00106	0.25460	0.77375	2	-0.00013	-0.03047	397.53598	958	0.41496	99.77586	-0.00031	0.48663	0.00224	0.15257	0.00255	0.33218
172	0.69556	1	-0.00056	-0.13697	1.77521	2	0.00220	0.53780	381.20051	936	0.40727	99.59917	0.00537	0.04535	0.00401	0.04059	-0.00137	1.00000
173	0.28706	1	-0.00065	-0.18346	1.09418	2	0.00091	0.25582	330.17538	932	0.35427	99.92764	0.00255	0.16020	0.00072	0.22792	-0.00183	1.00000
174	0.63277	1	0.00098	0.23627	0.41905	2	-0.00093	-0.22449	392.73225	950	0.41340	99.98822	-0.00225	0.92218	0.00012	0.65663	0.00236	0.33406
175	0.65072	1	0.00081	0.22941	0.56085	2	-0.00032	-0.09007	338.74814	964	0.35140	99.86066	-0.00090	0.55188	0.00139	0.27851	0.00229	0.33158
176	0.54859	1	-0.00068	-0.15406	1.59542	2	0.00177	0.40369	374.80832	856	0.43786	99.75037	0.00403	0.01832	0.00250	0.02129	-0.00154	0.66653
177	0.52787	1	0.00032	0.09108	0.74446	2	0.00008	0.02367	340.79069	964	0.35352	99.88526	0.00024	0.39604	0.00115	0.29337	0.00091	0.65416
178	0.58149	1	0.00014	0.03510	1.00376	2	0.00044	0.10639	378.22765	926	0.40845	99.85851	0.00106	0.27069	0.00141	0.20277	0.00035	0.66762
179	0.59329	1	-0.00003	-0.00689	1.17180	2	0.00065	0.14746	419.96971	950	0.44207	99.85943	0.00147	0.18782	0.00141	0.14406	-0.00007	0.65733
180	0.28158	1	0.00023	0.05913	0.41545	2	-0.00082	-0.21277	360.71293	940	0.38374	100.15364	-0.00213	0.83871	-0.00154	0.84901	0.00059	0.66802
181	2.17110	1	0.00362	0.85837	0.95862	2	0.00027	0.06505	398.21129	952	0.41829	99.07658	0.00066	0.31099	0.00923	0.00109	0.00858	0.33396
182	0.80508	1	0.00094	0.25941	0.75586	2	0.00007	0.01946	335.74766	926	0.36258	99.72112	0.00020	0.40158	0.00279	0.18505	0.00259	0.32545
183	0.85392	1	0.00082	0.20109	0.92096	2	0.00024	0.05872	390.67107	958	0.40780	99.74020	0.00059	0.32228	0.00260	0.10851	0.00201	0.32683
184	1.70514	1	0.00280	0.67325	0.78507	2	-0.00009	-0.02197	395.37154	958	0.41271	99.34873	-0.00022	0.47713	0.00651	0.00941	0.00673	0.33485
185	0.19037	1	-0.00036	-0.10357	0.70507	2	0.00003	0.00942	321.37865	930	0.34557	100.09415	0.00009	0.39317	-0.00094	0.54248	-0.00104	1.00000
186	0.31269	1	-0.00080	-0.18208	1.30894	2	0.00097	0.22251	421.34939	962	0.43799	99.95956	0.00222	0.09624	0.00040	0.19752	-0.00182	1.00000
187	0.08275	1	-0.00036	-0.13679	0.50922	2	-0.00004	-0.01653	254.88840	964	0.26441	100.15333	-0.00017	0.40347	-0.00153	0.59079	-0.00137	0.66881
188	0.31660	1	0.00020	0.05272	0.48388	2	-0.00065	-0.16904	362.81792	942	0.38516	100.11632	-0.00169	0.76020	-0.00116	0.76307	0.00053	0.33604
189	0.38720	1	0.00084	0.22334	0.11194	2	-0.00148	-0.39437	356.76086	946	0.37713	100.17102	-0.00395	0.99901	-0.00171	0.95366	0.00223	0.32693
190	1.00817	1	0.00108	0.24805	0.98254	2	0.00025	0.05794	416.92301	958	0.43520	99.69400	0.00058	0.30000	0.00306	0.04366	0.00248	0.32802
191	0.91613	1	0.00105	0.25598	0.85451	2	0.00009	0.02177	384.91608	944	0.40775	99.72225	0.00022	0.39139	0.00278	0.12594	0.00256	0.33911
192	0.76525	1	0.00111	0.26850	0.53475	2	-0.00065	-0.15777	393.85417	956	0.41198	99.88927	-0.00158	0.77584	0.00111	0.38891	0.00268	0.33050
193	0.40619	1	-0.00025	-0.05890	1.01418	2	0.00039	0.09286	384.97350	910	0.42305	99.96605	0.00093	0.25317	0.00034	0.30832	-0.00059	1.00000
194	0.44860	1	-0.00100	-0.22913	1.63230	2	0.00186	0.42645	390.69182	898	0.43507	99.80268	0.00425	0.04921	0.00197	0.08584	-0.00229	1.00000
195	0.18765	1	-0.00020	-0.05937	0.57905	2	-0.00022	-0.06591	319.53119	946	0.33777	100.12529	-0.00066	0.52594	-0.00125	0.69337	-0.00059	1.00000
196	0.65324	1	0.00035	0.08174	0.96216	2	0.00026	0.06084	407.46120	962	0.42356	99.85742	0.00061	0.31604	0.00143	0.18950	0.00082	0.33406
197	1.33675	1	0.00154	0.38553	1.16970	2	0.00085	0.21306	378.12117	952	0.39719	99.40141	0.00214	0.14723	0.00599	0.01119	0.00386	0.33158
198	0.33482	1	-0.00008	-0.02192	0.74501	2	0.00002	0.00475	356.06449	966	0.36860	100.01717	0.00005	0.42168	-0.00017	0.47851	-0.00022	0.65713
199	0.72738	1	0.00103	0.27259	0.51405	2	-0.00054	-0.14232	364.58331	966	0.37742	99.86973	-0.00143	0.70743	0.00130	0.35861	0.00273	0.33277
200	0.71393	1	0.00005	0.01198	1.30207	2	0.00114	0.28901	382.71501	970	0.39455	99.69901	0.00289	0.10030	0.00301	0.06168	0.00012	0.33851
201	0.96471	1	0.00106	0.25628	0.92128	2	0.00022	0.05329	395.01338	960	0.41147	99.69042	0.00053	0.32416	0.00310	0.08545	0.00256	0.33663
202	0.30189	1	-0.00050	-0.12340	0.99314	2	0.00047	0.11624	337.99119	838	0.40333	100.00716	0.00116	0.27535	-0.00007	0.40010	-0.00123	0.65931
203	0.72489	1	0.00018	0.04541	1.21909	2	0.00095	0.23704	382.14938	960	0.39807	99.71755	0.00237	0.15970	0.00282	0.08416	0.00045	0.66802
204	0.36723	1	0.00041	0.11628	0.40100	2	-0.00070	-0.19842	332.44893	940	0.35367	100.08214	-0.00199	0.75059	-0.00082	0.68455	0.00116	0.33436
205	0.22404	1	-0.00027	-0.06476	0.72164	2	-0.00029	-0.06938	398.11908	938	0.42443	100.13415	-0.00069	0.62743	-0.00134	0.80564	-0.00065	1.00000
206	0.82785	1	0.00050	0.11651	1.14828	2	0.00065	0.15149	408.21255	950	0.42970	99.73200	0.00152	0.18713	0.00268	0.07129	0.00117	0.33168
207	0.72659	1	0.00130	0.36487	0.29822	2	-0.00093	-0.26088	340.80091	960	0.35500	99.89601	-0.00262	0.89238	0.00104	0.43446	0.00365	0.33347
208	0.57921	1	0.00019	0.04523	0.96215	2	0.00027	0.06426	403.20084	958	0.42088	99.89051	0.00064	0.26653	0.00109	0.19416	0.00045	0.66376
209	0.43114	1	-0.00004	-0.01106	0.88154	2	0.00030	0.08123	354.32703	948	0.37376	99.92982	0.00081	0.33228	0.00070	0.32495	-0.00011	0.33554
210	0.66466	1	0.00139	0.39538	0.15248	2	-0.00128	-0.36542	323.41072	922	0.35077	99.97003	-0.00367	0.97822	0.00030	0.62842	0.00395	0.33010
211	1.56405	1	0.00262	0.65344	0.82496	2	0.00007	0.01773	354.72706	892	0.39768	99.32883	0.00018	0.40822	0.00671	0.02416	0.00653	0.33446
212	0.33393	1	0.00014	0.03259	0.57650	2	-0.00064	-0.14823	417.08338	966	0.43176	100.11564	-0.00148	0.80287	-0.00116	0.82198	0.00033	0.66376
213	0.92066	1	0.00142	0.37172	0.58877	2	-0.00039	-0.10344	349.27845	920	0.37965	99.73172	-0.00104	0.58109	0.00268	0.18584	0.00372	0.32040
214	0.39496	1	0.00047	0.13815	0.39008	2	-0.00066	-0.19236	327.40790	960	0.34105	100.05421	-0.00193	0.72762	-0.00054	0.63842	0.00138	0.66970
215	0.26850	1	0.00023	0.05460	0.38577	2	-0.00101	-0.24197	396.22335	952	0.41620	100.18738	-0.00242	0.93505	-0.00187	0.94673	0.00055	0.67277
216	0.36496	1	0.00027	0.06863	0.53438	2	-0.00059	-0.15041	356.25752	914	0.38978	100.08178	-0.00151	0.72604	-0.00082	0.69921	0.00069	0.66277
217	0.56993	1	0.00033	0.08956	0.81689	2	0.00018	0.04852	354.49413	962	0.36850	99.86192	0.00049	0.32099	0.00138	0.20020	0.00090	0.32980
218	0.49471	1	-0.00051	-0.12586	1.37828	2	0.00129	0.32142	384.90975	958	0.40178	99.80445	0.00321	0.09178	0.00196	0.10109	-0.00126	0.66574
219	0.09244	1	-0.00120	-0.32141	1.22501	2	0.00109	0.29400	354.81825	954	0.37193	100.02741	0.00293	0.14653	-0.00027	0.31891	-0.00321	1.00000
220	1.19359	1	0.00219	0.57362	0.41165	2	-0.00080	-0.21001	359.62367	944	0.38096	99.63640	-0.00211	0.81030	0.00364	0.15099	0.00574	0.33822
221	0.14520	1	-0.00096	-0.26443	1.13268	2	0.00092	0.25393	348.34505	964	0.36135	100.01050	0.00253	0.14485	-0.00010	0.27842	-0.00264	0.66644
222	0.43075	1	-0.00025	-0.05963	1.05450	2	0.00050	0.11854	390.36131	928	0.42065	99.94109	0.00118	0.26970	0.00059	0.30792	-0.00060	0.67208
223	0.11127	1	-0.00018	-0.05996	0.41345	2	-0.00046	-0.15230	278.21874	912	0.30506	100.21226	-0.00152	0.63426	-0.00212	0.78089	-0.00060	1.00000
224	0.47382	1	-0.00018	-0.04105	1.08127	2	0.00048	0.11137	407.98949	938	0.43496	99.92968	0.00111	0.19020	0.00070	0.19604	-0.00041	1.00000
225	0.38312	1	0.00010	0.02596	0.68438	2	-0.00025	-0.06243	381.85159	960	0.39776	100.03647	-0.00062	0.54752	-0.00036	0.55752	0.00026	0.32594
226	0.80979	1	0.00018	0.04224	1.37059	2	0.00120	0.28043	398.09147	934	0.42622	99.67732	0.00281	0.09723	0.00323	0.04050	0.00042	0.32634
227	2.14870	1	0.00436	1.00249	0.93377	2	0.00019	0.04367	345.74921	804	0.43004	98.95384	0.00044	0.35723	0.01046	0.00119	0.01002	0.32921
228	1.09443	1	0.00186	0.62390	0.47178	2	-0.00028	-0.09350	281.17484	946	0.29722	99.46960	-0.00094	0.58109	0.00530	0.07525	0.00624	0.33495
229	1.02595	1	0.00111	0.24840	1.00972	2	0.00027	0.05940	419.14692	938	0.44685	99.69221	0.00060	0.29347	0.00308	0.03475	0.00248	0.32762
230	1.31487	1	0.00176	0.43757	0.97683	2	0.00040	0.09905	377.10979	940	0.40118	99.46337	0.00099	0.25416	0.00537	0.02208	0.00438	0.33208
231	0.94546	1	0.00095	0.22414	0.96970	2	0.00027	0.06333	408.63764	962	0.42478	99.71253	0.00063	0.31277	0.00287	0.07485	0.00224	0.32990
232	0.17320	1	-0.00093	-0.23352	1.05107	2	0.00068	0.17220	334.60002	844	0.39645	100.06132	0.00172	0.24347	-0.00061	0.46000	-0.00234	1.00000
233	0.36137	1	-0.00001	-0.00359	0.73403	2	0.00002	0.00432	351.14171	966	0.36350	99.99927	0.00004	0.38485	0.00001	0.40436	-0.00004	0.66337
234	0.56625	1	0.00033	0.07580	0.83050	2	-0.00007	-0.01587	411.45012	956	0.43039	99.94007	-0.00016	0.50644	0.00060	0.38446	0.00076	0.66525
235	0.20401	1	-0.00083	-0.20495	1.12504	2	0.00073	0.18006	379.79991	942	0.40318	100.02489	0.00180	0.16891	-0.00025	0.34040	-0.00205	0.65980
236	0.38607	1	0.00043	0.13221	0.41432	2	-0.00055	-0.16860	301.45553	926	0.32555	100.03638	-0.00169	0.67485	-0.00036	0.56238	0.00132	0.33010
237	1.47858	1	0.00214	0.56589	0.95677	2	0.00047	0.12357	353.59430	940	0.37616	99.31054	0.00124	0.24693	0.00689	0.01861	0.00566	0.32663
238	0.79828	1	0.00071	0.15188	0.95559	2	0.00006	0.01217	426.55918	916	0.46568	99.83594	0.00012	0.41861	0.00164	0.12366	0.00152	0.33000
239	0.39618	1	-0.00023	-0.06104	0.96010	2	0.00051	0.13454	333.84552	888	0.37595	99.92650	0.00134	0.26525	0.00073	0.29089	-0.00061	0.66525
240	1.05664	1	0.00181	0.46995	0.46723	2	-0.00069	-0.17799	367.72300	956	0.38465	99.70804	-0.00179	0.75980	0.00292	0.20168	0.00470	0.32297
241	0.28968	1	0.00032	0.08150	0.35300	2	-0.00101	-0.25473	373.24867	944	0.39539	100.17323	-0.00255	0.91851	-0.00173	0.91257	0.00082	0.33307
242	1.04083	1	0.00046	0.10614	1.53723	2	0.00154	0.35579	410.11336	952	0.43079	99.53807	0.00356	0.03416	0.00462	0.00574	0.00106	0.33673
243	0.87426	1	0.00069	0.16706	1.06556	2	0.00056	0.13613	392.53528	960	0.40889	99.69681	0.00136	0.20099	0.00303	0.06653	0.00167	0.33485
244	0.98155	1	0.00162	0.44996	0.48024	2	-0.00054	-0.14886	346.16295	966	0.35835	99.69890	-0.00150	0.67455	0.00301	0.19663	0.00450	0.33713
245	0.43899	1	-0.00082	-0.18466	1.55199	2	0.00150	0.33602	420.98481	946	0.44502	99.84864	0.00335	0.03455	0.00151	0.06624	-0.00185	1.00000
246	0.78724	1	0.00093	0.22023	0.71035	2	-0.00029	-0.06886	408.65405	972	0.42043	99.84862	-0.00069	0.63970	0.00151	0.29089	0.00220	0.32960
247	0.51743	1	-0.00000	-0.00076	0.99446	2	0.00067	0.19018	330.51137	942	0.35086	99.81058	0.00190	0.24307	0.00189	0.21931	-0.00001	0.67574

248	1.17095	1	0.00230	0.54594	0.33839	2	-0.00117	-0.27776	386.82941	920	0.42047	99.73182	-0.00279	0.96030	0.00268	0.27297	0.00546	0.33703
249	2.74387	1	0.00442	1.13221	1.21937	2	0.00101	0.25887	371.10184	964	0.38496	98.60892	0.00262	0.11990	0.01391	0.00010	0.01132	0.33762
250	2.32081	1	0.00263	0.71221	2.01454	2	0.00288	0.77852	348.89646	958	0.36419	98.50928	0.00784	0.01881	0.01491	0.00000	0.00712	0.33495
251	0.34936	1	0.00043	0.10313	0.37403	2	-0.00105	-0.25119	398.57452	956	0.41692	100.14806	-0.00251	0.95564	-0.00148	0.93238	0.00103	0.34089
252	0.66025	1	0.00098	0.25339	0.45016	2	-0.00073	-0.18825	367.63382	950	0.38698	99.93486	-0.00189	0.80683	0.00065	0.49990	0.00253	0.33030
253	0.24220	1	0.00020	0.05257	0.35953	2	-0.00093	-0.24227	363.30478	948	0.38323	100.18970	-0.00242	0.88733	-0.00190	0.90050	0.00053	0.66733
254	0.17146	1	-0.00037	-0.08809	0.70599	2	-0.00028	-0.06762	398.90678	960	0.41553	100.15572	-0.00068	0.55050	-0.00156	0.76644	-0.00088	0.66119
255	0.27197	1	0.00003	0.00728	0.54539	2	-0.00048	-0.12740	356.17629	940	0.37891	100.12012	-0.00127	0.65594	-0.00120	0.71703	0.00007	0.32941
256	0.47830	1	0.00069	0.16659	0.37704	2	-0.00100	-0.24363	397.70768	964	0.41256	100.07704	-0.00244	0.92475	-0.00077	0.80436	0.00167	0.32356
257	0.66046	1	0.00049	0.12262	0.86476	2	0.00013	0.03325	376.96201	936	0.40274	99.84413	0.00033	0.36505	0.00156	0.21406	0.00123	0.67287
258	0.15568	1	-0.00005	-0.01269	0.41350	2	-0.00077	-0.20408	368.50167	970	0.37990	100.21677	-0.00204	0.79891	-0.00217	0.90257	-0.00013	0.67426
259	0.69502	1	-0.00084	-0.20948	1.96718	2	0.00268	0.66671	380.47169	952	0.39966	99.54278	0.00665	0.01198	0.00457	0.00772	-0.00209	1.00000
260	0.42540	1	-0.00028	-0.07751	1.06234	2	0.00077	0.21416	345.60854	962	0.35926	99.86335	0.00214	0.17079	0.00137	0.17762	-0.00078	1.00000
261	0.29111	1	-0.00117	-0.28325	1.53652	2	0.00165	0.40098	385.57237	936	0.41194	99.88227	0.00400	0.04584	0.00118	0.10535	-0.00283	1.00000
262	0.10332	1	-0.00089	-0.24284	0.96497	2	0.00055	0.15006	334.37833	914	0.36584	100.09277	0.00150	0.22782	-0.00093	0.45941	-0.00243	0.67297
263	1.34563	1	0.00179	0.40804	1.01000	2	0.00031	0.07145	409.33054	936	0.43732	99.52051	0.00072	0.30158	0.00479	0.01356	0.00408	0.33634
264	0.83754	1	0.00084	0.20004	0.89864	2	0.00015	0.03635	388.22856	932	0.41655	99.76361	0.00036	0.36624	0.00236	0.13327	0.00200	0.33089
265	0.29405	1	-0.00010	-0.02828	0.68671	2	-0.00007	-0.01839	341.51820	954	0.35799	100.04667	-0.00018	0.45356	-0.00047	0.51950	-0.00028	1.00000
266	0.17538	1	-0.00072	-0.18030	0.99118	2	0.00043	0.10656	379.72478	944	0.40225	100.07375	0.00106	0.27089	-0.00074	0.48317	-0.00180	1.00000
267	1.00950	1	0.00147	0.33883	0.68466	2	-0.00041	-0.09513	408.74361	944	0.43299	99.75630	-0.00095	0.65198	0.00244	0.13208	0.00339	0.33426
268	0.74615	1	0.00073	0.18866	0.79185	2	0.00004	0.01012	376.24274	972	0.38708	99.80121	0.00010	0.39337	0.00199	0.19059	0.00189	0.32465
269	0.32655	1	0.00006	0.01852	0.60360	2	-0.00009	-0.02862	307.99068	956	0.32217	100.01010	-0.00029	0.43198	-0.00010	0.44366	0.00019	0.66911
270	0.72472	1	0.00073	0.19044	0.79463	2	0.00007	0.01913	348.98828	914	0.38183	99.79043	0.00019	0.42495	0.00210	0.22396	0.00190	0.32822
271	0.14834	1	-0.00212	-0.59478	2.02466	2	0.00304	0.85174	335.48556	942	0.35614	99.74304	0.00847	0.00832	0.00257	0.02931	-0.00595	1.00000
272	1.19042	1	0.00159	0.38688	0.87587	2	0.00013	0.03198	392.31130	960	0.40866	99.58114	0.00032	0.35238	0.00419	0.04446	0.00387	0.32901
273	0.23700	1	-0.00015	-0.03239	0.65445	2	-0.00057	-0.12527	445.26161	976	0.45621	100.15766	-0.00125	0.82436	-0.00158	0.93554	-0.00032	0.67683
274	0.75660	1	-0.00013	-0.02805	1.53378	2	0.00146	0.32266	421.58409	936	0.45041	99.70538	0.00323	0.02139	0.00295	0.00743	-0.00028	0.66644
275	0.17682	1	0.00024	0.06624	0.21290	2	-0.00114	-0.32022	340.51927	952	0.35769	100.25398	-0.00320	0.94703	-0.00254	0.96059	0.00066	0.33525
276	0.55065	1	0.00110	0.32572	0.14523	2	-0.00119	-0.35158	324.92731	962	0.33776	100.02586	-0.00353	0.99287	-0.00026	0.72307	0.00326	0.33604
277	0.30407	1	-0.00039	-0.09432	0.93154	2	0.00027	0.06596	366.54439	896	0.40909	100.02836	0.00066	0.29822	-0.00028	0.43545	-0.00094	1.00000
278	1.11413	1	0.00189	0.44969	0.50506	2	-0.00075	-0.17800	401.83517	960	0.41858	99.72832	-0.00179	0.82752	0.00272	0.19465	0.00450	0.34218
279	0.40777	1	0.00061	0.15089	0.31339	2	-0.00110	-0.27331	389.97678	966	0.40370	100.12242	-0.00274	0.94970	-0.00122	0.86871	0.00151	0.33188
280	0.41544	1	0.00007	0.01460	0.79152	2	-0.00026	-0.05822	377.19281	844	0.44691	100.04362	-0.00058	0.61327	-0.00044	0.62624	0.00015	0.66178
281	0.37863	1	-0.00025	-0.06031	0.96028	2	0.00034	0.08308	377.65680	928	0.40696	99.97724	0.00083	0.30495	0.00023	0.36178	-0.00060	1.00000
282	0.62393	1	0.00075	0.19021	0.58996	2	-0.00046	-0.11758	369.07609	934	0.39516	99.92737	-0.00118	0.68287	0.00073	0.44802	0.00190	0.32901
283	0.20125	1	-0.00071	-0.16362	1.02082	2	0.00035	0.07976	400.84951	918	0.43666	100.08387	0.00080	0.29248	-0.00084	0.56178	-0.00164	1.00000
284	0.68108	1	0.00130	0.34612	0.23624	2	-0.00118	-0.31321	353.08692	938	0.37643	99.96708	-0.00314	0.96564	0.00033	0.60337	0.00346	0.33188
285	0.18494	1	-0.00087	-0.20496	1.10467	2	0.00061	0.14467	386.65034	912	0.42396	100.06028	0.00144	0.21535	-0.00060	0.45297	-0.00205	1.00000
286	0.22861	1	-0.00026	-0.06147	0.71659	2	-0.00031	-0.07320	399.05693	936	0.42634	100.13467	-0.00073	0.62861	-0.00135	0.80119	-0.00061	1.00000
287	0.52785	1	0.00036	0.09876	0.72332	2	-0.00002	-0.00661	344.20106	938	0.36695	99.90785	-0.00007	0.41752	0.00092	0.31703	0.00099	0.33356
288	0.76311	1	0.00122	0.30589	0.49837	2	-0.00072	-0.18012	357.29519	896	0.39877	99.87423	-0.00181	0.79327	0.00126	0.37238	0.00306	0.33010
289	0.48192	1	-0.00006	-0.01324	1.00289	2	0.00024	0.05299	425.80615	948	0.44916	99.96024	0.00053	0.31792	0.00040	0.32149	-0.00013	0.66129
290	1.21268	1	0.00060	0.13152	1.76281	2	0.00201	0.44440	414.62083	920	0.45067	99.42408	0.00445	0.00634	0.00576	0.00030	0.00132	0.66356
291	0.59647	1	-0.00004	-0.01040	1.18548	2	0.00075	0.17442	409.63092	960	0.42670	99.83597	0.00174	0.14733	0.00164	0.11228	-0.00010	0.66644
292	1.28686	1	0.00199	0.46779	0.71727	2	-0.00030	-0.06954	407.54047	960	0.42452	99.60175	-0.00070	0.61901	0.00398	0.06238	0.00468	0.34188
293	0.37663	1	0.00033	0.08175	0.48822	2	-0.00072	-0.17779	392.74317	968	0.40573	100.09604	-0.00178	0.82356	-0.00096	0.77950	0.00082	0.66525
294	0.54466	1	0.00019	0.05072	0.89217	2	0.00034	0.09280	348.76763	940	0.37103	99.85648	0.00093	0.27089	0.00144	0.21050	0.00051	0.66455
295	0.34123	1	-0.00017	-0.03882	0.84455	2	-0.00006	-0.01466	415.41464	952	0.43636	100.05348	-0.00015	0.44495	-0.00053	0.55663	-0.00039	0.66604
296	0.11247	1	-0.00205	-0.63525	1.96651	2	0.00295	0.91580	311.56065	970	0.32120	99.71945	0.00910	0.01188	0.00281	0.03475	-0.00635	1.00000
297	0.51475	1	0.00052	0.12873	0.58174	2	-0.00051	-0.12580	383.57888	954	0.40207	99.99707	-0.00126	0.70663	0.00003	0.55277	0.00129	0.33020
298	1.06818	1	0.00180	0.47590	0.50407	2	-0.00057	-0.15057	356.68565	946	0.37705	99.67468	-0.00151	0.71069	0.00325	0.18465	0.00476	0.33455
299	1.42661	1	0.00255	0.67926	0.57466	2	-0.00040	-0.10608	345.20088	926	0.37279	99.42682	-0.00107	0.63248	0.00573	0.06673	0.00679	0.33832
300	0.74492	1	0.00011	0.02998	1.30008	2	0.00122	0.31868	363.88938	956	0.38064	99.65134	0.00319	0.08455	0.00349	0.03921	0.00030	0.66792
301	1.00294	1	0.00096	0.23112	1.10730	2	0.00065	0.15695	377.21871	908	0.41544	99.61193	0.00157	0.34723	0.00388	0.13554	0.00231	0.66891
302	0.96843	1	-0.00005	-0.01169	1.82412	2	0.00250	0.64913	348.12522	908	0.38340	99.36256	0.00649	0.01644	0.00637	0.00287	-0.00012	0.33297
303	0.80963	1	0.00071	0.24206	0.92044	2	0.00078	0.26509	269.87923	920	0.29335	99.49285	0.00266	0.11644	0.00507	0.03366	0.00242	0.33723
304	2.08400	1	0.00435	1.22514	0.51637	2	-0.00047	-0.13231	301.24185	858	0.35110	98.90717	-0.00134	0.59762	0.01093	0.01535	0.01225	0.32772
305	0.55848	1	0.00119	0.30219	0.12749	2	-0.00151	-0.38503	370.48492	944	0.39246	100.08283	-0.00386	0.99822	-0.00083	0.86129	0.00302	0.32634
306	0.27772	1	-0.00002	-0.00594	0.58887	2	-0.00023	-0.06681	316.57401	922	0.34336	100.07275	-0.00067	0.48535	-0.00073	0.54921	-0.00006	0.66099
307	1.66995	1	0.00301	0.90943	0.58877	2	-0.00016	-0.04706	307.94192	938	0.32830	99.13763	-0.00047	0.47683	0.00862	0.03594	0.00909	0.33426
308	1.39103	1	0.00183	0.42944	1.10494	2	0.00061	0.14376	386.44520	914	0.42281	99.42680	0.00144	0.22000	0.00573	0.01149	0.00429	0.33198
309	0.27261	1	-0.00089	-0.23015	1.31690	2	0.00120	0.30867	379.05204	978	0.38758	99.92147	0.00308	0.10386	0.00079	0.18564	-0.00230	1.00000
310	0.48334	1	-0.00005	-0.01349	0.97770	2	0.00054	0.14533	357.85146	972	0.36816	99.86816	0.00145	0.24960	0.00132	0.22970	-0.00013	0.34386
311	0.80460	1	-0.00063	-0.14988	2.03920	2	0.00266	0.63183	406.63875	970	0.41922	99.51806	0.00631	0.00554	0.00482	0.00198	-0.00150	0.66564
312	0.50400	1	-0.00056	-0.13928	1.44375	2	0.00142	0.35323	390.02248	970	0.40209	99.78605	0.00353	0.06614	0.00214	0.07109	-0.00139	0.67168
313	0.56168	1	0.00072	0.19417	0.49978	2	-0.00055	-0.14900	346.93854	940	0.36908	99.95483	-0.00149	0.69089	0.00045	0.48465	0.00194	0.33743
314	0.48592	1	-0.00028	-0.08138	1.15000	2	0.00110	0.32119	315.19096	926	0.34038	99.76019	0.00321	0.10792	0.00240	0.10317	-0.00081	0.67337
315	0.96492	1	0.00129	0.34208	0.74172	2	-0.00003	-0.00763	353.74877	938	0.37713	99.66555	-0.00008	0.42446	0.00334	0.12554	0.00342	0.32703
316	0.36772	1	0.00070	0.17454	0.17477	2	-0.00140	-0.35238	381.63876	956	0.39920	100.17784	-0.00353	0.99099	-0.00178	0.94822	0.00175	0.32861
317	0.24277	1	-0.00019	-0.05358	0.67073	2	-0.00008	-0.02203	342.24255	970	0.35283	100.07561	-0.00022	0.46713	-0.00076	0.58010	-0.00054	0.66495
318	0.25996	1	-0.00109	-0.26500	1.42951	2	0.00140	0.34114	384.35830	938	0.40976	99.92386	0.00340	0.05624	0.00076	0.13644	-0.00265	1.00000
319	0.25928	1	-0.00251	-0.59613	2.61750	2	0.00399	0.94668	403.54438	960	0.42036	99.64945	0.00941	0.00010	0.00351	0.00277	-0.00596	1.00000
320	0.39718	1	0.00012	0.03479	0.68218	2	0.00003	0.00930	322.11175	964	0.33414	99.95591	0.00009	0.38010	0.00044	0.34574	0.00035	0.66228
321	0.90651	1	0.00118	0.26821	0.90705	2	0.00009	0.02041	343.47833	784	0.43811	99.71138	0.00020	0.39000	0.00289	0.10059	0.00268	0.33366
322	0.03588	1	-0.00036	-0.11862	0.42167	2	-0.00045	-0.14691	278.62105	912	0.30551	100.26553	-0.00147	0.61307	-0.00266	0.85545	-0.00119	1.00000
323	0.24572	1	0.00016	0.04992	0.38336	2	-0.00061	-0.18816	306.79814	944	0.32500	100.13825	-0.00188	0.72743	-0.00138	0.72772	0.00050	0.33188
324	1.38340	1	0.00189	0.42784	1.00418	2	0.00029	0.06482	413.62513	940	0.44003	99.50734	0.00065	0.29228	0.00493	0.00980	0.00428	0.33327
325	0.37398	1	-0.00038	-0.09300	1.04505	2	0.00055	0.13534	370.08974	908	0.40759	99.95766	0.00135	0.23129	0.00042	0.29287	-0.00093	1.00000
326	0.15636	1	-0.00020	-0.06373	0.51327	2	-0.00028	-0.08969	290.08363	916	0.31669	100.15342	-0.00090	0.53495	-0.00153	0.69723	-0.00064	1.00000
327	1.30324	1	0.00150	0.36102	1.14585	2	0.00072	0.17492	396.26623	962	0.41192	99.46406	0.00176	0.16347	0.00536	0.01347	0.00361	0.32743
328	1.22085	1	0.00196	0.44525	0.65904	2	-0.00049	-0.11208	415.67578	948	0.43848	99.66683	-0.00113	0.74931	0.00333	0.08584	0.00445	0.32832
329	0.79947	1	0.00045	0.11108	1.14618	2	0.00078	0.19111	376.18497	930	0.40450	99.69781	0.00191	0.15733	0.00302	0.06564	0.00111	0.33703
330	0.18366	1	-0.00063	-0.15841	0.91078	2	0.00026	0.06490	361.46406	902	0.40074	100.09351	0.00065	0.34376	-0.00094	0.56574	-0.00158	0.66228
331	2.33481	1	0.00484	1.31894	0.33344	2	-0.00091	-0.24862	336.76845	928	0.36290	98.92968	-0.00252	0.85564	0.01070	0.00832	0.01319	0.33475
332	0.32022	1	-0.00156	-0.36368	1.93614	2	0.00243	0.56660	412.00555	962	0.42828	99.79709	0.00565	0.00911	0.00203	0.02812	-0.00364	1.00000

333	0.41485	1	0.00028	0.07590	0.58901	2	−0.00037	−0.09747	357.95439	954	0.37521	100.02156	−0.00098	0.57564	−0.00022	0.51248	0.00076	0.65881
334	0.35961	1	0.00041	0.10573	0.38901	2	−0.00089	−0.22720	374.82637	956	0.39208	100.12147	−0.00227	0.86337	−0.00121	0.81366	0.00106	0.66950
335	0.37981	1	−0.00074	−0.19323	1.36648	2	0.00135	0.35080	368.12093	960	0.38346	99.84244	0.00350	0.07851	0.00158	0.10703	−0.00193	1.00000
336	0.23405	1	−0.00089	−0.23979	1.20121	2	0.00107	0.28786	341.82179	918	0.37235	99.95194	0.00287	0.10455	0.00048	0.18683	−0.00240	0.66267
337	0.12721	1	−0.00181	−0.43497	1.76395	2	0.00216	0.51887	388.05542	932	0.41637	99.91610	0.00517	0.02129	0.00084	0.09584	−0.00435	1.00000
338	0.84295	1	0.00009	0.01998	1.50842	2	0.00144	0.33265	415.71488	962	0.43214	99.64736	0.00333	0.04950	0.00353	0.01832	0.00020	0.33168
339	0.89376	1	0.00037	0.08347	1.37427	2	0.00113	0.25500	415.50413	944	0.44015	99.66153	0.00255	0.09653	0.00338	0.02634	0.00083	0.66832
340	0.46672	1	0.00070	0.20672	0.41438	2	−0.00069	−0.20380	268.77601	798	0.33681	99.99707	−0.00204	0.70495	0.00003	0.55782	0.00207	0.34248
341	0.30998	1	−0.00117	−0.35002	1.58280	2	0.00206	0.61369	321.38569	962	0.33408	99.73633	0.00612	0.03545	0.00264	0.05950	−0.00350	0.66238
342	2.14781	1	0.00377	0.90131	0.80054	2	−0.00007	−0.01584	393.23968	948	0.41481	99.11452	−0.00016	0.47683	0.00885	0.00208	0.00901	0.33723
343	1.24420	1	0.00252	0.66811	0.28828	2	−0.00108	−0.28567	347.07335	922	0.37644	99.61756	−0.00288	0.92851	0.00382	0.17970	0.00668	0.32911
344	0.63533	1	0.00020	0.04750	1.07941	2	0.00060	0.14370	359.45269	862	0.41700	99.80881	0.00144	0.23653	0.00191	0.15535	0.00047	0.66267
345	0.47397	1	−0.00052	−0.12250	1.35528	2	0.00115	0.27039	399.05479	936	0.42634	99.85211	0.00270	0.07693	0.00148	0.09970	−0.00122	0.65990
346	0.27510	1	−0.00050	−0.14150	0.93370	2	0.00058	0.16296	303.63263	860	0.35306	99.97854	0.00163	0.24723	0.00021	0.32743	−0.00142	0.66000
347	0.76870	1	0.00120	0.26856	0.56756	2	−0.00079	−0.17807	382.60690	858	0.44593	99.90951	−0.00179	0.88901	0.00090	0.43525	0.00269	0.33446
348	1.61660	1	0.00228	0.54059	1.04797	2	0.00047	0.11260	402.80230	962	0.41871	99.34681	0.00113	0.22960	0.00653	0.00564	0.00541	0.33099
349	1.11155	1	0.00185	0.48731	0.54122	2	−0.00049	−0.13010	356.94976	942	0.37893	99.64278	−0.00131	0.65119	0.00357	0.13446	0.00487	0.33317
350	0.54781	1	0.00043	0.11461	0.69459	2	−0.00014	−0.03664	365.49163	966	0.37836	99.92202	−0.00037	0.49485	0.00078	0.35574	0.00115	0.66713
351	0.61182	1	0.00095	0.25793	0.38345	2	−0.00079	−0.21531	352.39350	958	0.36784	99.95737	−0.00216	0.86881	0.00043	0.56545	0.00258	0.33129
352	0.63421	1	−0.00024	−0.07145	1.38351	2	0.00164	0.49608	312.86592	950	0.32933	99.57538	0.00496	0.04842	0.00425	0.03079	−0.00071	0.66347
353	0.80926	1	0.00144	0.35019	0.37763	2	−0.00104	−0.25234	380.85934	926	0.41130	99.90214	−0.00253	0.93129	0.00098	0.48713	0.00350	0.33545
354	0.65725	1	0.00042	0.10261	0.90785	2	0.00019	0.04738	392.43386	956	0.41050	99.85001	0.00047	0.35950	0.00150	0.22040	0.00103	0.33248
355	0.69507	1	0.00068	0.15703	0.76087	2	−0.00024	−0.05598	419.01513	964	0.43466	99.89895	−0.00056	0.58515	0.00101	0.32614	0.00157	0.33663
356	0.29004	1	−0.00030	−0.07466	0.82543	2	0.00003	0.00717	340.16548	836	0.40690	100.06749	0.00007	0.45436	−0.00067	0.57356	−0.00075	0.67554
357	0.43240	1	−0.00010	−0.02378	0.94143	2	0.00022	0.05241	403.70325	956	0.42228	99.97136	0.00052	0.34802	0.00029	0.37762	−0.00024	0.66941
358	0.96570	1	0.00022	0.05302	1.61208	2	0.00181	0.44002	386.59170	946	0.40866	99.50696	0.00440	0.02743	0.00493	0.00673	0.00053	0.66594
359	0.60557	1	0.00061	0.15902	0.65105	2	−0.00026	−0.06881	371.67122	966	0.38475	99.90980	−0.00069	0.55356	0.00090	0.36446	0.00159	0.33248
360	0.99577	1	0.00153	0.35151	0.66070	2	−0.00049	−0.11228	399.09677	920	0.43380	99.76077	−0.00113	0.72376	0.00239	0.18604	0.00352	0.32762
361	0.26268	1	−0.00022	−0.05266	0.74586	2	−0.00020	−0.04736	400.95523	962	0.41679	100.10002	−0.00047	0.53267	−0.00100	0.67485	−0.00053	0.66198
362	0.84142	1	0.00114	0.28122	0.61914	2	−0.00042	−0.10444	395.62107	976	0.40535	99.82323	−0.00105	0.66198	0.00177	0.27307	0.00281	0.33000
363	0.16457	1	−0.00034	−0.08256	0.65085	2	−0.00043	−0.10326	362.86105	880	0.41234	100.18582	−0.00103	0.66129	−0.00186	0.85059	−0.00083	1.00000
364	0.90492	1	0.00126	0.35034	0.70237	2	−0.00004	−0.01010	320.65886	894	0.35868	99.65976	−0.00010	0.43366	0.00340	0.11277	0.00350	0.33129
365	0.80667	1	0.00116	0.26558	0.63719	2	−0.00057	−0.12999	385.85165	886	0.43550	99.86441	−0.00130	0.77059	0.00136	0.32505	0.00266	0.33089
366	0.66979	1	0.00044	0.10556	0.92882	2	0.00022	0.05167	378.86724	904	0.41910	99.84276	0.00052	0.37604	0.00157	0.22881	0.00106	0.33297
367	0.77294	1	0.00001	0.00146	1.44434	2	0.00139	0.32624	394.01506	930	0.42367	99.67230	0.00326	0.08307	0.00328	0.04178	0.00001	0.67356
368	1.16981	1	0.00219	0.53514	0.38850	2	−0.00098	−0.23981	384.45435	944	0.40726	99.70467	−0.00241	0.91851	0.00295	0.18782	0.00535	0.33020
369	0.27881	1	−0.00136	−0.33118	1.65765	2	0.00197	0.47919	373.89615	910	0.41087	99.85199	0.00478	0.02772	0.00148	0.06525	−0.00331	1.00000
370	1.36148	1	0.00108	0.25693	1.62780	2	0.00187	0.44429	381.82513	912	0.41867	99.29878	0.00445	0.02535	0.00701	0.00089	0.00257	0.66396
371	0.84539	1	0.00010	0.02240	1.49660	2	0.00144	0.31958	407.22599	908	0.44849	99.65802	0.00320	0.03485	0.00342	0.00990	0.00022	0.66921
372	0.45061	1	−0.00057	−0.13677	1.33347	2	0.00118	0.27985	383.46210	914	0.41954	99.85691	0.00279	0.13921	0.00143	0.18950	−0.00137	0.66604
